# From waste to resource: circular valorization of spent mushroom substrate into biochar and advanced materials

**DOI:** 10.3389/fchem.2026.1768384

**Published:** 2026-04-14

**Authors:** Anagha Sunil, Julia L. Shamshina

**Affiliations:** 1 Department of Plant and Soil Science, Fiber and Biopolymer Research Institute, Texas Tech University, Lubbock, TX, United States; 2 Department of Chemistry and Biochemistry, Texas Tech University, Lubbock, TX, United States

**Keywords:** biochar, biofuels, composting, materials, spent mushroom substrate (SMS), vermitechnology

## Abstract

Spent mushroom substrate (SMS), also known as spent mushroom compost, is the material that is left over after a mushroom crop has been harvested. They typically contain materials like straw, sawdust, or manure, and are rich in mycelium and leftover nutrients. SMS represents a reliable as well as sustainable lignocellulosic feedstock to produce biofuels, hydrochars and value-added products. Evidently, as an abundant waste in the agricultural field, SMS contains high levels of organic matter, making it a promising candidate for conversion into biofuels such as bioethanol, biogas and biochar by different thermochemical and biochemical processes. Hydrothermal carbonization can convert SMS into hydrochars, which can be then used for various applications including but not limited to carbon-based materials and adsorbents. In biofuel production, SMS has shown that it is valuable in fermentation into bioethanol with enzyme action. Due to its lignocellulosic nature, SMS can be further processed into durable materials with exceptional adsorptive properties, can be used in water treatment, pollution control, and soil remediation. Furthermore, the conversion of SMS into biofuels and materials offers a well-round solution for waste management, reducing environmental pollution, promoting circular economies, and supporting the development of renewable energy systems. This review examines the diverse uses of SMS, focusing on its potential as a biochar, biofuel and material precursor, and highlights the technological challenges and opportunities for optimizing the conversion processes.

## 1 Introduction

Spent mushroom substrate (SMS) is the residual material remaining after mushroom cultivation, typically composed of lignocellulosic agricultural by-products such as straw, sawdust, manure, or corn cobs interwoven with fungal mycelium. Across numerous studies, it is consistently reported that approximately 5 kg of wet SMS is generated for every kilogram of fresh mushrooms harvested, (Lam et al., 2019) resulting in an estimated 242 million tons of SMS produced globally in 2022 (Martín et al., 2023). In the United States alone, about 406 million metric tons are produced annually, with Pennsylvania, particularly Chester County (Kennett Square) accounting for roughly 69% of national production (Marín-Benito et al., 2016; Subchapter, 2026). The sheer scale of SMS generation creates both environmental management challenges and economic opportunities for valorization.

Disposal of SMS remains a significant burden for mushroom growers, who must often pay landfill or transport fees ranging from $10 to $50 per tonne (Hoover et al., 2025). The bulky, high-moisture nature of SMS (60%–80% water content) makes it difficult and expensive to handle, while limited recycling options and appropriate processing infrastructure exacerbate the problem (Hanafi et al., 2018). Uncontrolled disposal, such as dumping or incineration, can lead to eutrophication, water contamination, and greenhouse gas emissions, particularly methane. As landfill emissions are projected to account for nearly 10% of global greenhouse gas output by 2025, many regions have begun restricting organic waste disposal and encouraging alternative reuse strategies (Wan Mahari et al., 2020).

Lignocellulosic SMS is also difficult to digest anaerobically because of its rigid structure and high lignin content. Lignin, partially degraded cellulose/hemicellulose, and fungal-derived polymers form a dense, crosslinked structure that enzymes and microbes cannot easily penetrate, slowing the conversion of biomass into biogas and making pretreatment or alternative valorization pathways necessary for efficient utilization (Zheng et al., 2014). Moreover, disposal is not cost-neutral: landfill tipping fees vary by region and facility, and transportation costs are elevated due to the high moisture content and low bulk density of SMS.

Landfills contribute significantly to global warming by emitting biogas, primarily methane. According to the International Solid Waste Association (ISWA), landfill emissions are projected to account for 10% of global greenhouse gas emissions by 2025 if current trends continue (Hoover et al., 2025). In response to these environmental concerns—including methane release and leachate contamination—many regions have introduced restrictions or outright bans on organic waste disposal in landfills (Hanafi et al., 2018). In Canada, for example, Nova Scotia, Prince Edward Island, and Ontario have already enacted organic waste bans (Hénault-Ethier et al., 2017). Internationally, South Korea has demonstrated the effectiveness of such measures, achieving a 90% recycling rate through mandatory composting and volume-based waste fees (Lee E. et al., 2024). These developments reflect a growing global shift toward sustainable waste management and the reduction of landfill-related methane emissions.

Amid increasing concern about landfill-derived methane, a pressing question is whether organic waste such as SMS can be repurposed as a renewable heat or energy source instead of being discarded. However, with a typical moisture content of 60%–80%, (Grimm et al., 2021) SMS is heavy and voluminous, increasing transportation and storage costs while significantly lowering its calorific value. This high-water content severely limits the efficiency of thermal processing methods such as combustion or gasification. To make SMS suitable for energy recovery, it must first be dried to approximately 10% moisture, a step that incurs additional costs ranging from $30 to $100 per ton, depending on energy prices and local drying infrastructure.

It is therefore evident that SMS, generated at massive scale, poses significant economic and environmental burdens, and that its sustainable management requires intentional valorization into value-added products and materials rather than disposal or low-efficiency energy use. To frame these challenges and opportunities, this review systematically examines the literature on SMS valorization, with emphasis on application-driven pathways where chemical and materials-based approaches are most impactful.

**FIGURE 1:**
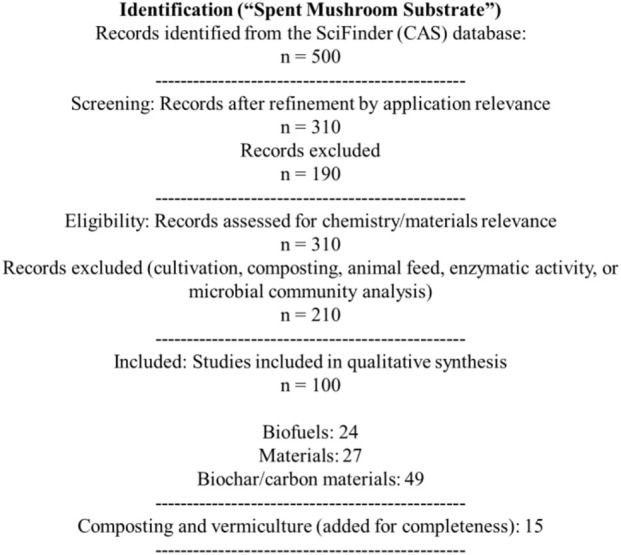
PRISMA-style flow diagram illustrating the literature search and selection process for spent mushroom substrate (SMS) valorization studies. A SciFinder (CAS) search conducted on February 15 identified 500 records. Following refinement by application relevance, 310 records were screened. Of these, 210 studies were excluded due to a primary focus on cultivation, composting, agronomic use, animal feed, enzymatic activity, or microbial community analysis. The remaining 100 studies were included in the qualitative synthesis and categorized into biofuels (24), materials (27), and biochar/carbon-based materials (49). Studies on composting and vermiculture provided for completeness (15).

A comprehensive search conducted in the SciFinder (CAS) database on 15 February 2026 retrieved approximately 500 references, including both original research articles and reviews, using the term “spent mushroom substrates.” Following refinement by application relevance, 310 records were retained for initial screening. Of these, 100 studies in which SMS serves as a chemically transformable feedstock were categorized according to their dominant valorization route into biofuels (24 references), materials (27 references), and biochar and carbon-based materials (49 references), Figure 1.

**FIGURE 2:**
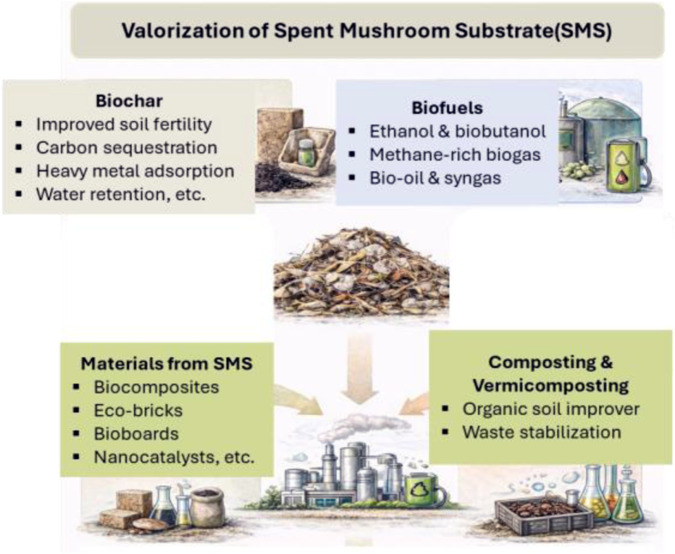
Major valorization pathways for spent mushroom substrate (SMS), including biochar production, biofuels generation, materials development, and agronomic reuse through composting and vermicomposting. These routes illustrate the diverse strategies for converting SMS into value-added products within circular bioeconomy frameworks.

The remaining 210 studies focus primarily on cultivation systems, production practices, animal feed, mulch, and related agronomic or microbiological investigations. Many of these works represent well-established biological reuse routes for SMS that have been widely documented elsewhere. Historically, the majority of SMS research has emphasized lower-value agricultural reuse pathways, particularly aerobic composting and vermitechnology (vermicomposting or vermiculture-based biotransformation), which consistently emerge as practical “organics-to-products” strategies for converting SMS into marketable soil amendments while reducing disposal burdens and environmental risks associated with uncontrolled stockpiling. A brief overview of vermiculture-based biotransformation is therefore also provided in the end of review, for completeness, see Figure 2 for major SMS valorization routes.

The distribution of studies identified in the present analysis (biofuels, materials, and biochar) reflects a growing research emphasis on engineered materials and carbon-rich platforms. While agronomic reuse pathways such as composting and vermiculture remain important and widely practiced, the increasing attention to materials-oriented applications underscores the emerging role of SMS as a versatile feedstock for advanced materials development and circular bioeconomy strategies.

## 2 Composition

Before exploring upscaling solutions for spent mushroom substrate (SMS), it is important to understand the nature of this waste. SMS originates from the mushroom cultivation medium, which is primarily composed of lignocellulosic agricultural by-products such as sawdust, straw, hay, cottonseed hulls, corn cobs, etc. In other words, SMS itself is a lignocellulosic biomass, typically containing 40%–60% organic matter. This composition gives it inherent potential for value-added applications yet also present challenges due to its heterogeneity and partial degradation.

Because SMS is not a uniform material, but rather a formulation- and species-dependent residue, its chemical composition, physical structure, and residual biopolymer content vary substantially across production systems. For this reason, this review is intentionally organized to (i) define what “waste mushroom-based biopolymers” practically encompass in real SMS streams and mushroom-derived residues, and (ii) map how compositional differences translate into downstream processing behavior and material performance. This feedstock-centered framing is not ancillary but foundational: without it, cross-study comparisons of conversion yield, porosity development, surface chemistry, adsorption capacity, or catalytic activity become misleading, as the starting materials are not equivalent across reports.

Table 1 provides an overview of commonly used substrates for mushroom cultivation, highlighting their composition, characteristics, and suitability for different mushroom species. The most widely used materials include agricultural lignocellulosic byproducts, such as wheat or barley straw, hardwood sawdust, etc. Straw-based substrates are inexpensive, easy to pasteurize, and commonly used for oyster and Enokitake (*Flammulina velutipes*) mushrooms, although they have low nitrogen content (often <0.5%) (Wieser et al., 2020). Hardwood sawdust, often enriched with wheat bran, mimics the natural habitat of wood-decaying fungi like shiitake and reishi but requires sterilization and has slower colonization rates.

**TABLE 1:** Overview of commonly used substrates for mushroom cultivation.

Material	Mushrooms cultivated	Composition (%)	Characteristics	Ref
100% Wheat Or Barley Straw: chopped into short pieces and usually pasteurized with hot water or lime	Oyster (all varieties), enoki, wine cap (garden giant), shaggy mane, and some *Agaricus* (button) species	Wheat • Starch: 58 • Non-starch polysacch.: 13 • Proteins: 11 Barley straw • Cellulose: 37–40 • Hemicellulose: 29–35 • Lignin: 15–20	• Very inexpensive • Widely available • Provides a fibrous structure with good air flow • High moisture retention • Can be effectively pasteurized (no need for full sterilization) • Low nitrogen content (often <0.5% N)	Wieser et al. (2020)
Hardwood Sawdust: oak or beech, often sourced as compressed wood pellets plus a supplement like wheat bran (∼10–20% by weight) for extra nutrients	Shiitake, lion’s mane, maitake, reishi, king oyster, nameko, and chestnut	Hardwood • Cellulose: 37–50 • Hemicellulose: 17–30 • Lignin: 17–30	• Mimics a fungus’ natural decaying-wood habitat • Support high yields over multiple flushes • Ample nutrition • Requires thorough sterilization • Colonization is relatively slow	Le Floch et al. (2015)
Straw & Hardwood Sawdust Blend Material A 50:50 or similar mix of chopped straw and hardwood sawdust	Oyster, wine cap, shiitake	Sawdust • Cellulose: 48 • Lignin: 33	• Balances slow-degrading lignin and readily consumed cellulose • Widely available • Low-cost	Le Floch et al. (2015), Wieser et al. (2020)
Master’s Mix: (50% Hardwood Sawdust +50% Soy Hulls) A highly nutritive 1:1 blend of hardwood sawdust and soybean hulls (often both in pellet form), hydrated to ∼60% moisture and sterilized before use	Oyster (e.g., blue, golden, pink), lion’s mane, chestnut, reishi	Hardwood • Cellulose: 37–50 • Hemicellulose: 17–30 • Lignin: 17–30 Soy hulls • Cellulose: 40.6 • Hemicellulose: 33.8 • Lignin: 13.1	• Richer in nitrogen and minerals • Higher contamination risk and cost • Soy hulls may be less accessible in some regions	Le Floch et al. (2015), Robles Barros et al. (2020)
Coco Coir & Vermiculite (“CVG” Mix) Coco coir (fibrous coconut husk) mixed roughly 1:1 with vermiculite, plus a small amount of gypsum (5%–10% gypsum by volume is common). Typically prepared by soaking in boiling water (bucket tek) which both hydrates and pasteurizes the substrate	Golden halo, oyster, and *Panaeolus*	Coco coir • Cellulose: 36–43 • Hemicellulose: 0.25 • Lignin: 32–45	• Easy and clean to work with • The coir provides excellent water retention and allows good airflow • Vermiculite aids in even distribution of moisture • Gypsum helps buffer pH and add minerals to mycelium	Mahmud et al. (2023)
Manure-Based Compost Composted horse or cow manure, often mixed with straw, bedding, and gypsum and allowed to partially decompose	White/brown button mushrooms, cremini portobello, golden halo	• Cellulose: 6–7 • Hemicellulose: 6–7 • Lignin: 18–19	• Nutrient rich and high yielding • Preparation is labor-intensive • High contamination risk	Geng et al. (2024)
Spent Coffee Grounds Used coffee grounds (usually collected fresh from cafés or kitchens). Best used fresh (within 24 h of brewing)	*Pleurotus* species	• Cellulose: 12 • Hemicellulose: 39 • Lignin: 23	• Highly sustainable • No sterilization needed • Contain residual sugars, proteins, and a favorable carbon/nitrogen ratio 17 • Collection logistics can be a challenge for larger projects	Chai et al. (2021)
Cardboard Plain corrugated cardboard (undyed, non-glossy) soaked in hot water. Typically layered or sandwich-stacked with mushroom spawn	*Pleurotus* species	ND	• Essentially free and abundant • Easy preparation • Extremely low nutritional value • Dries out fast	Oys et al. (2020)
Wood Chips Coarse hardwood wood chips or mulch (often from landscape waste or woodchippers). Common species used include oak, maple, poplar, alder, etc. Usually utilized in outdoor beds or mulch gardens rather than indoor bags, since chips colonize slowly. Often combined with straw or sawdust to speed colonization	Wine cap, turkey tail	Hardwood • Cellulose: 37–50 • Hemicellulose: 17–30 • Lignin: 17–30	• Low cost • Long-lastingContain complex nutrients and lignin • Retails moisture well • High maintenance • Slow first yield	Le Floch et al. (2015)
Logs (Outdoor Hardwood Logs) Sections of freshly-cut hardwood logs (commonly oak, beech, maple, sweetgum, etc.), usually 3–8 inches in diameter and around 1 m long. Logs are inoculated by drilling holes and inserting wooden dowel spawn or sawdust spawn, then sealing the holes with wax	Shiitake, oysters, lion’s mane, reishi, maitake, wood ear, and turkey tail	Hardwood • Cellulose: 37–50 • Hemicellulose: 17–30 • Lignin: 17–30	• Low maintenance and longevity • Less control • Not suitable for rapid or high-volume production	Le Floch et al. (2015)
Brown Rice Flour & Vermiculite (PF Tek Cakes) A mix of brown rice flour (BRF), vermiculite, and water – typically 1 part BRF: 2 parts medium vermiculite to 1 part water by volume. This is packed into small jars and steam-sterilized (or pressure-cooked) to create a sterile “cake”	Golden halo	Brown Rice Flour • Cellulose: 3.8	• Ingredients are cheap and easy to obtain • The method is low-tech and reliable; the vermiculite creates a moist, airy matrix for mycelium while the rice flour provides a balanced nutrient source • Limited yield and scale	Fan et al. (2023)

Blended substrates, such as 50:50 straw and sawdust mixes or the highly nutritive “Master’s Mix” (equal parts hardwood sawdust and soy hulls), balance nutrient availability and degradation rates, supporting high yields for species like lion’s mane and oyster. Alternatives such as coco coir with vermiculite (CVG mix), coffee grounds, and cardboard offer low-cost or recycled options with varying nutrient profiles and ease of preparation. Manure-based composts are rich in nutrients and used predominantly for button mushrooms but come with higher contamination risks and labor demands. Less conventional or outdoor substrates include wood chips and whole hardwood logs, which support species such as wine cap, turkey tail, and shiitake but require longer colonization and yield times. Lastly, the PF Tek method using brown rice flour and vermiculite provides a beginner-friendly, small-scale approach for golden halo mushrooms. These cultivation practices underscore both the diversity of mushroom substrates and the resulting lignocellulosic complexity of SMS. This diversity directly governs the physicochemical properties of SMS and, consequently, its behavior during thermochemical conversion, biochemical processing, and materials fabrication, reinforcing the necessity of a feedstock-informed framework for evaluating SMS valorization pathways.

Given its lignocellulosic composition and high organic matter content, SMS offers significant potential for value-added applications beyond traditional use. Historically employed as animal feed (Aiduang et al., 2025; Baptista et al., 2023; Boontiam et al., 2020; Liu et al., 2015; Yuan et al., 2022) or a soil amendment. SMS is rich in cellulose, hemicellulose, and lignin, key components that make it suitable for advanced material and energy recovery processes. Technologies such as hydrothermal carbonization, anaerobic digestion, and fermentation can convert SMS into biofuels like biogas and bioethanol. It can also be processed into biochar, supporting both renewable energy production and carbon sequestration. Beyond energy uses, SMS serves as a precursor for biocomposites, activated carbon, and adsorbents applied in water treatment, pollution mitigation, and soil remediation. These valorization routes reinforce circular economy principles by reducing waste and enabling sustainable technological solutions.

## 3 Biochar from SMS

### 3.1 Biochar fundamentals

Biochar, a carbon-rich, porous solid produced through pyrolysis of biomass under limited oxygen (typically 300 °C–700 °C), has gained prominence as a multifunctional material for carbon sequestration, pollutant removal, and soil conditioning. It is engineered through controlled pyrolysis to achieve specific surface and chemical functionalities.

**FIGURE 3:**
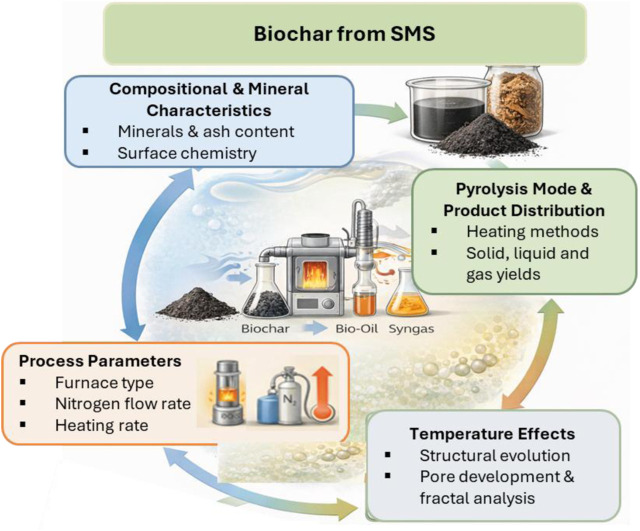
Schematic overview of factors influencing biochar production from spent mushroom substrate (SMS), including feedstock composition, pyrolysis conditions, temperature-dependent structural evolution, and resulting product distribution (biochar, bio-oil, and syngas).

Spent mushroom substrate (SMS), the residual medium left after mushroom cultivation, is an abundant and under-utilized feedstock for biochar production. Each ton of mushrooms generates roughly three to five tons of SMS, ensuring a steady, renewable feedstock for thermochemical valorization. Converting SMS into biochar transforms this liability into a value-added product, reducing disposal costs and offering potential revenue streams for growers. Its composition, rich in lignocellulosic residues, organic matter, and mineral nutrients, makes SMS particularly suitable for high-performance biochar synthesis. Partial biological decomposition during mushroom growth introduces microstructural openings and nitrogen-containing compounds which facilitate pore development and nitrogen incorporation during subsequent carbonization. The major factors governing SMS-to-biochar conversion and the resulting product properties are summarized schematically in Figure 3.

### 3.2 Compositional and mineral characteristics of SMS

The biochemical composition of SMS plays a decisive role in determining the textural and functional characteristics of the derived biochar. The relative proportions of cellulose, hemicellulose, and lignin in SMS vary with substrate and fungal species, dictating the resulting biochar’s structure and reactivity. The lignin-to-cellulose ratio particularly affects carbon yield, fixed-carbon content, and structural stability after pyrolysis (Jayakumar et al., 2023).

Feedstocks with higher lignin content, such as hardwood residues, tend to form aromatic, graphitic carbon networks that impart rigidity and long-term stability (Xu X. et al., 2024). Hardwood tissues, dense and rich in vascular vessels, produce high-surface-area, microporous biochars due to controlled devolatilization and carbon condensation. The aromatic carbon frameworks formed during pyrolysis enhance both thermal stability and fixed-carbon yield, yielding durable materials suited for activated-carbon and carbon-storage applications (Suota et al., 2021; Wu et al., 2024).

In contrast, herbaceous residues such as wheat bran are richer in proteins, hemicellulose, and lipids, (Li et al., 2023) generating biochars with abundant heteroatom (O- and N-) functionalities such as–COOH, –OH, and–NH_2_ groups. These heteroatom sites facilitate surface complexation, cation exchange, and cation–π interactions with metal ions. SMS, which contains both lignocellulosic residues and fungal proteins, combines these features, producing a structurally stable yet chemically active biochar.

The SMS-derived biochar (SMSBC) exhibited a BET surface area of ∼184 m^2^ g^−1^ and a well-developed mesoporous network that enabled hydroxyapatite (HAP) loading. The resulting HAP/SMSBC composite displayed a surface area of 452.6 m^2^ g^−1^, uniform nanoparticle distribution, and significantly enhanced Pb^2+^ and Cd^2+^ adsorption capacities, as well as improved pH buffering stability in acidic environments.

To further optimize these complementary attributes, several studies explored hybridization of SMS with other lignocellulosic residues. Thus, hybrid biochars combining wheat bran and sawdust integrate the complementary properties of each feedstock. Bran provides surface-active functional groups, while sawdust contributes mechanical stability and porous structure. The resulting blended biochar achieved synergistically improved metal sorption performance for Pb^2+^ and Cd^2+^ ions, demonstrating that feedstock mixing can be strategically employed to balance reactivity and structural resilience (Fan et al., 2024).

Thus, the intrinsic chemical composition of the biomass governs the carbonization pathway and the surface chemistry of the resulting biochar. Lignin-rich precursors favor aromaticity and microporosity, while protein- and polysaccharide-rich materials yield heteroatom-doped, reactive surfaces traits that can be tailored through feedstock selection to meet specific functional objectives.

Most spent mushroom substrate (SMS) formulations incorporate nutrient and structural additives to optimize fungal growth, substrate porosity, and pH balance. Among these, calcium-containing minerals such as gypsum (CaSO_4_) and lime (CaO) are commonly added to the culture medium to supply essential nutrients and regulate acidity (Chang et al., 2019). These minerals neutralize organic acids produced by fungal metabolism during cultivation and sterilization, while also preventing substrate compaction, improving aeration and water movement, and maintaining the pH within the optimal 6–7 range for mushroom development.

Although introduced primarily to support mycelial growth, these inorganic additives exert a lasting influence on the physicochemical properties of the resulting biochar (Fan et al., 2024). During pyrolysis, mineral compounds such as calcium sulfate are thermally transformed and immobilized within the carbon matrix, yielding Ca-enriched biochars with distinctive reactivity (Zhang et al., 2014). Such materials exhibit enhanced adsorption capacities for heavy metals including Cd^2+^ and Cu^2+^, (Jin et al., 2021) attributed to increased cation-exchange capacity, modified surface charge, and the generation of reactive mineral sites that serve as stable binding centers for divalent cations. PretreatThe incorporation of calcium into the biochar lattice also promotes precipitation or ion-exchange reactions, thereby improving efficiency in water purification, soil remediation, and other environmental detoxification applications (Li et al., 2017).

In addition to mineral additives, most SMS formulations contain organic supplements such as bran, molasses, or other carbohydrate sources that act as nutrient enhancers during mushroom cultivation. These organic excipients significantly affect the chemical evolution of the biochar matrix. During pyrolysis, they decompose into oxygen-rich intermediates, contributing to the formation of surface functional groups, notably carboxyl (–COOH), hydroxyl (–OH), and carbonyl (C=O) moieties (Zhang P. et al., 2022; Zhang et al., 2024)which further enhance the pollutant removal performance, compared to that of “pristine” biochar (Zhang P. et al., 2022). These groups enhance the affinity of biochar surfaces for both inorganic and organic contaminants, facilitating complexation, hydrogen bonding, and π–π interactions with species such as metal ions, pharmaceuticals, and dyes.

Woody and lignocellulosic components of SMS also contain mineral nutrients absorbed during plant growth, many of which persist through pyrolysis and become concentrated in the ash fraction, influencing chemical reactivity, stability, and soil-amendment potential. For example, sawdust from mahogany, teak, and sappan wood pyrolyzed at 250 °C–350 °C exhibited distinct elemental profiles by X-ray fluorescence (XRF): calcium oxide (CaO) accounted for 51%–72% of total ash, potassium oxide (K_2_O) for 9%–23%, and phosphorus pentoxide (P_2_O_5_) for 2%–10% (Rahmat et al., 2023). Sappan-derived biochar showed the highest CaO content, mahogany the highest K_2_O, and teak the highest P_2_O_5_; additionally, teak biochar contained 15%–20% silica (SiO_2_), while sappan biochar was enriched in 6%–8% iron oxide (Fe_2_O_3_).

These compositional variations directly affect biochar alkalinity, nutrient release, and catalytic potential. The dominance of alkaline cations (Ca^2+^, Mg^2+^, K^+^) confers strong buffering and liming capacity, enabling such biochars to neutralize acidic soils and enhance nutrient availability. The coexistence of P- and Fe-bearing oxides further supports phosphate retention and contaminant immobilization, extending their environmental applications beyond pH correction.

Because SMS retains residual Ca, K, and P from both its original lignocellulosic substrate and added fungal nutrients, its biochar inherits similar mineral-induced alkalinity and nutrient functionality. Understanding this combined organic–inorganic matrix is key for tailoring SMS biochar properties.

### 3.3 Pyrolysis mode and product distribution

The product distribution of the pyrolysis process depends strongly on operational parameters such as temperature, pressure, and residence time (Tomczyk et al., 2020). Fast pyrolysis, conducted at 400 °C–600 °C under vacuum to atmospheric pressure with a short residence time of seconds, primarily yields bio-oil (75%), with lower outputs of synthetic gas (13%) and biochar (12%). In contrast, slow pyrolysis (biocarbonization), performed at 350 °C–800 °C under atmospheric pressure for residence times ranging from minutes to hours, produces a more balanced distribution: 30% bio-oil, 35% synthetic gas, and 35% biochar. Slow pyrolysis is particularly suited to SMS due to its partially decomposed lignocellulosic matrix.

### 3.4 Temperature effects on devolatilization dynamics and pore morphology

#### 3.4.1 Structural evolution with temperature

While feedstock composition dictates the chemical foundation of biochar, pyrolysis temperature governs the structural evolution and degree of carbon ordering. At higher temperatures (≥500 °C), thermal degradation promotes aromatization and graphitization, leading to higher surface area, porosity, pH, and ash content, but also to a loss of oxygen-containing groups that decreases the cation exchange capacity (CEC) (Tomczyk et al., 2020). These transformations increase chemical stability and resistance to oxidation, making high-temperature biochars more suitable for carbon sequestration. Conversely, low-temperature biochars (300 °C–400 °C) retain more polar surface functionalities (–OH, –COOH), enhancing reactivity and metal-binding capacity, which are advantageous for soil and environmental applications.

Conversely, low-temperature biochars (300 °C–400 °C) retain more polar surface functionalities (–OH, –COOH), enhancing reactivity and metal-binding capacity, which are advantageous for soil and environmental applications. In summary, temperature determines the balance between structural condensation and chemical functionality, shaping the biochar’s performance envelope.

#### 3.4.2 Pore development and fractal analysis

A computed tomography (CT)-based study of coarse Chinese fir wood provided a detailed mechanistic view of how temperature, devolatilization dynamics, and pore morphology are interrelated during fluidized-bed pyrolysis (Chen et al., 2018). The dense vascular structure of coarse wood resulted in a longer devolatilization period than fine biomass (e.g., sawdust), but increasing the temperature from 450 °C to 700 °C caused a near-linear reduction in devolatilization time. Higher thermal input accelerated volatile release, generating microcracks and channels that reduced diffusion resistance and facilitated mass transfer, a positive feedback process that promoted progressive pore development.

At intermediate temperatures (∼600 °C) and moderate residence times (5–7 min), complete devolatilization produced a well-connected mesoporous network (2–10 nm). The BET surface area and total pore volume increased markedly, from 33.9 m^2^ g^−1^ to 0.02 cm^3^ g^−1^ at 500 °C to 214.6 m^2^ g^−1^ and 0.08 cm^3^ g^−1^ at 700 °C, respectively (Chen et al., 2018). Excessive heating, however, led to pore shrinkage and collapse due to localized softening and melting of carbonized particles. Thus, the pore architecture effectively records the thermal history of the biomass: rapid devolatilization at high temperatures yields tortuous, irregular pore networks, whereas slower, lower-temperature pyrolysis produces more uniform but less porous structures. Fractal analysis of the resulting chars showed that increasing temperature and devolatilization rate correlate with higher surface (D_1_) and volume (D_2_) fractal dimensions, reflecting greater structural roughness and complexity.

#### 3.4.3 Optimization and trade-offs

Temperature governs the balance between carbon ordering and surface functionalization. Optimal pyrolysis conditions depend on the intended application: moderate pyrolysis (500 °C–600 °C) yields biochars with both accessible mesoporosity and active surface groups, whereas extreme temperatures (>700 °C) produce stable, graphitic materials with diminished chemical reactivity. Feedstock composition and pyrolysis temperature act as complementary determinants of biochar performance. Composition dictates the chemical blueprint (lignin vs. protein content, mineral inclusions), while temperature controls the extent of carbon reorganization and pore evolution. By aligning these parameters through feedstock blending, controlled heating, and post-treatment, biochar structure and surface chemistry can be precisely tuned to meet distinct goals such as carbon sequestration, catalytic support, soil conditioning, or heavy-metal remediation.

### 3.5 Process parameters affecting SMS biochar

This section analyzes representative studies on biochar production from spent mushroom substrate (SMS) in a parameter-by-parameter manner, beginning with furnace type and reactor configuration, followed by nitrogen flow conditions, pyrolysis temperature, heating rate, residence time, and finally the resulting adsorption performance. Because generalized claims regarding “SMS biochar” are potentially misleading when feedstock- and process-specific context is not provided, Table 2 consolidates and critically compares available experimental literature, highlighting the combined influence of substrate composition, mushroom species, and thermochemical processing conditions on pollutant removal efficiency. We can see from this table that performance outcomes are strongly dependent on feedstock identity and processing history; unlike biochar derived from a single, well-defined biomass, SMS-based biochar exhibits substantial variability due to differences in cultivation substrates, fungal metabolism, residual mineral content, and retained mycelial biomass.

**TABLE 2:** Studies on biochar production from SMS, highlighting the influence of substrate composition, mushroom species, and pyrolysis conditions on pollutant removal performance.

Mushroom species	SMS	Oven type	Temperature (°C)	Heating rate (C/min)	Residence time (min)	Removal of	Activity^a^ (mg/g)	Ref
ND	Sawdust and middlings	Tubular, N_2_ atmosphere	400–800	ND	15–75	Pb^2+^ Cd^2+^	90.58 25.35	Zhang et al. (2014)
*A. bisporus*						Cd^2+^ Cu^2+^ Pb^2+^	64.80 68.10 55.20	Boulanger et al. (2024), Ristić et al. (2023)
*A. auricula*						Cd^2+^ Cr^3+^	114.60 118.00	Wang et al. (2023)
*L. edodes*						Pb^2+^	398.00	Zhang et al. (2023)
*P. ostreatus*						Cd^2+^ Pb^2+^	55.95 326.00	Han et al. (2023), Zhang et al. (2023)
*G. lucidum*						Cd^2+^ Pb^2+^	23.81–75.82 141.59–262.76	Han et al. (2023)
Compost only						Cu^2+^ Pb^2+^ Zn^2+^	52.60–364.00 564.00 332.00	Han et al. (2023), Kojić et al. (2022)
*Djulis Chenopodium formosanum*	Not specified	Circulation, N_2_ atmosphere	500–800	10	15–60	Methylene blue	41.16	Zhang et al. (2022b)
ND	Saw dust, wheat bran	Tubular	100–500	10	ND	Pb^2+^ Cd^2+^	866.25 133.71	Zhang et al. (2024)
*Pleurotus ostreatus*	Not specified	Muffle furnace	550	5	360	Aflatoxin	ND	K et al. (2022)
ND	Not specified	ND	700–800	10	120	NaCl	8.38	​
*L. edodes*	Not specified	Muffle furnace	500	15	180	Tetracycline	37.95 31.89 (Fe^2+^) 25.36. (Fe^0^) 9.63 (Fe^3+^)	Alves et al. (2022)
ND	Oak wood, cotton seed hull, wheat bran, corn flour and gypsum	Teflon lined autoclave	500–700	ND	ND	Rhodamine B, Amoxicillin, and Cefixime	ND	Lin et al. (2021)
*Pleurotus ostreatus*	Birch wood sawdust -second flush	Tubular furnace	20–500	5	120	Orange-16 dye	519.00	Liu et al. (2021)
ND	Sawdust, wheat bran, calcium sulfate and excipients	Muffle furnace	500	5	240	Cu, Zn, oxytetracycline, enrofloxacin	ND	Jiang et al. (2024)
*Auricularia auricula*	Not specified	Not specified	600	10	360	Cd^2+^ Cr^4+^	22.00 23.70	Sarfraz et al. (2020)
*C. indica*	Not specified	Autoclave, regular oven	60–121	ND	2880	Iron^3+^	ND	Wu et al. (2019)
ND	Pinewood sawdust	Automatic vertical autoclave	180	5	120	Carbon	1.25	Wenhao et al. (2019)
*Pleurotus pulmonarius*	Birch sawdust and wheat bran	Tube furnace	500–700	10	60	NO_3_^−^	0.73	Li et al. (2017)
*L. edodes*	Sawdust:wheat bran:calcium sulfate:excipients	Tube furnace	500	5	240	Composite	ND	Sewu et al. (2019)
ND	Not specified	Not specified	180	ND	60	Formaldehyde	4.80	Corral-Bobadilla et al. (2019)
*Oyster mushrooms*	Not specified	Not specified	500	ND	360	Cd^2+^	1.41	Wang et al. (2023)
*Lentinula edodes*	Wheat and rice bran, eucalyptus sawdust, and water	Not specified	250–450, 600	ND	60	Ethinylestradiol and progestron	ND	Zhang et al. (2023)
*Auricularia auricula*	Not specified	Tube furnace	500	3	120	Methylene blue	25.51	Han et al. (2023)
*Agaricus bisporus*	Not specified	Not specified	500	5	60	Pb^2+^ Cd^2+^	297.00 131.00	Kojić et al. (2022)
*Ganoderma lucidum*	Not specified	Not specified	350–750	15	180	Cationic dyes MG and ST	9,388.04 3,871.48	Zhang et al. (2022b)
*L. edodes*	Not specified	Not specified	500	ND	ND	Cd^2+^	ND	Li et al. (2022)
*L. edodes*	eucalyptus and grassy straw	Not specified	200	10	120	CO_2_	8.25	Alves et al. (2022)
Not specified	Not specified	Not specified	450	ND	240	N_2_O	ND	Xu et al. (2022)
Not specified	Not specified	Not specified	400	ND	120	NH_3_	6.60	Lin et al. (2021)
*Auricularia auricula*	Not specified	Muffle furnace	350–600	10	240	Cu^2+^	52.60–65.60	Alves et al. (2022)
*Agaricus bisporus*	Not specified	Tubular	300–600	10	240	Microcystin	0.13	Liu et al. (2021)
*prTremella fuciformis*	Not specified	Stainless steel furnace	700	10	180	Phosphorous	0.05	Sarfraz et al. (2020)
*P. ostreatus*	Bran, corn cob and rice straw	Muffle furnace	300–700	ND	120	Pb^2+^	326.00	Wu et al. (2019)
*Shiitake*	Bran, corn cob and rice straw	Muffle furnace	300–700	ND	120	Pb^2+^	398.00	Wu et al. (2019)
*Tremella fuciformis*	Not specified	Steel furnace, N_2_ atmosphere	500	10	120	Cd^2+^	97.00	Wenhao et al. (2019)
Not specified	Not specified	Tube reactor, N_2_ atmosphere	800	ND	120	Crystal violet dye	1057.00	Sewu et al. (2019)
*Agaricus bisporus*	Straw, poultry manure, horse manure, gypsum, and urea	Bio reactor	ND	ND	ND	Pb^2+^ Fe^2+^ Fe^3+^	87.00 94.00 97.00	Corral-Bobadilla et al. (2019)
*Pleurotus ostreatus*	Bran, corn cob and rice straw	Muffle furnace	300–700	ND	120	Cd^2+^	24.94	Wu et al. (2019)
Not specified	Not specified	Stainless steel furnace, N_2_ atmosphere	500	10	60	Crystal violet dye	ND	Pan et al. (2021)
*Agaricus bisporus*	Bagasse, corncob and sawdust	Tube furnace, N_2_ atmosphere	550–750	5	30	Nitrogen	0.60	(Kumar et al., 2021)
*Lentinus edodes*	Hardwood, bran and lime	Vertical pyrolysis furnace, N_2_ atmosphere	300–800	10	20	ND	ND	Chen et al. (2022)
*Flammulina velutipes* *L. edodes*	Not specified	Biomass carbonizer, N_2_ atmosphere	300–700	10	120	ND	ND	Zhao et al. (2019)
*L. edodes*	Ca-rich SMS	Tube furnace, N_2_ atmosphere	400–600	10	180	Malachite Green oxalate (MG) dye	9,388	Zhang et al. (2022b)
*Agaricus bisporus*	Composted straw and gypsum	Muffle furnace, N_2_ atmosphere	350–750	10	180	Cu^2+^ Zn^2+^ Cd^2+^	11.60–68.10 16.90–55.20 17.20–64.80	Zhang et al. (2020)
SMS from 6 mushroom types (incl. *Pleurotus*, *Auricularia*)	Not specified	Muffle furnace, N_2_ atmosphere	300–700	10	240	Cu^2+^	52.60–65.60	Jin et al. (2021)

^a^ Activity: refers to the effectiveness of biochar in adsorbing or removing specific contaminants, expressed as adsorption capacity contaminant (mg) per g biochar.

Overall, the reviewed studies employ a wide variety of SMS feedstocks, including sawdust-based substrates, wheat bran-supplemented formulations, agricultural residues, and nutrient-enriched cultivation media, reflecting both the diversity of mushroom production practices and the resulting complexity of SMS-derived biochar properties. The most studied mushroom species include *Pleurotus ostreatus*, *Lentinus edodes*, *Agaricus bisporus*, and *Auricularia auricula*, each contributing distinct biological residues that affect biochar functionality. Pyrolysis conditions vary significantly across studies: temperatures range from 200 °C to 800 °C, residence times from 15 to 360 min, and heating rates from 3 °C to 15 °C/min. Muffle and tubular furnaces are the most frequently used equipment types. It appears that biochar performance is highly tunable, depending on the biological input (mushroom species), substrate formulation, and pyrolysis parameters. These findings support the strategic design of SMS-based biochars for targeted environmental remediation applications.

#### 3.5.1 Furnace type

The type of furnace used significantly influences the preparation and properties of SMS biochar. Tubular furnaces are widely applied due to their ability to uniformly distribute heat and maintain controlled conditions during pyrolysis (Xu et al., 2024b). For smaller-scale experiments, muffle furnaces provide precise temperature control and are cost-effective (Jin et al., 2021). However, muffle furnaces provide inconsistent oxygen exclusion. Air exposure causes oxidation or ash formation. Biochar produced in the muffle furnace is found to contain 70% more ash, 78% more fixed carbon, and 63% less volatile matter than the biochar produced in a traditional retort kiln (Paz-Ferreiro et al., 2018). This means producing biochar with consistent carbon yield may not be achieved.

To produce biochar in a muffle furnace, the precursor biomasses are packed into ceramic crucibles and covered to limit their exposure to oxygen These crucibles were then placed into the furnace and pyrolyzed at desired temperatures. Although sealing the crucible can effectively prevent exposure to oxygen, creating a completely air-tight system is not advisable. Thermal expansion of gases during heating may generate internal pressure that can crack or even fracture the ceramic vessel, akin to how rigid refractories can spall under thermal stress. An alternative is to include wood charcoal inside the furnace as an oxygen scavenger. Charcoal, being nearly pure carbon, reacts with residual oxygen to form CO/CO_2_-consuming oxygen without combusting itself. It should be replaced every 3–4 pyrolysis cycles to ensure continued effectiveness and to prevent burning or smoke emissions (Lamichhanae et al., 2025). The exact pyrolysis time depended on the feedstock. Stainless-steel reactors, as well as specialized setups like Teflon-lined autoclaves, offer the flexibility to manipulate both temperature and pressure. Such configurations ensure versatility in biochar production for various applications (Wu et al., 2024).

#### 3.5.2 Nitrogen flow rate

Inert gases like nitrogen are used during pyrolysis to prevent oxidation. Nitrogen flow rates typically range from 200 to 2000 cm^3^/min, ensuring a stable, oxygen-free environment that facilitates the carbonization process. Higher flow rates are often associated with better-quality biochar due to uniform carbon deposition (Morgan et al., 2025). At high gas flows there is relatively little accumulation of pyrolysis vapors in the space between the biomass particles, and the intra-particle residence time of vapors is proportional to particle size. Hence an increase in yield and uniformity is observed with particle size at higher gas flows (Somerville and Deev, 2020). For instance, studies reveal that a flow rate of 300–500 cm^3^/min is optimal for achieving high adsorptive properties in biochar (Fan et al., 2024).

#### 3.5.3 Temperature range, heating rate, and residence time

The pyrolysis temperature profoundly affects the biochar’s properties. At lower temperatures (200 °C–400 °C), the biochar retains more volatile compounds, which may be desirable for specific adsorptive applications. However, higher temperatures (500 °C–800 °C) lead to increased surface area and porosity, making the material ideal for heavy metal removal and dye adsorption (Boulanger et al., 2024). For instance, biochar prepared at 700 °C has shown significant efficacy in adsorbing contaminants like rhodamine B and heavy metals (Zhao et al., 2024).

The heating rate during pyrolysis typically ranges between 5 °C and 15 °C/min. A moderate heating rate of 10 °C/min is commonly adopted to ensure uniform carbonization without causing structural distortions in the biochar (Almeida Moreira et al., 2024). Faster heating rates might lead to incomplete conversion of the substrate, while slower rates enhance the material’s stability and adsorptive potential.

Residence time is another critical factor in biochar preparation. Holding the SMS at the target temperature for prolonged periods, ranging from 15 min to 6 h, impacts the thermal decomposition of the substrate and the quality of the end product. For example, a 4-h residence time at 550 °C in a muffle furnace resulted in biochar with excellent adsorption capacity for aflatoxins and heavy metals (Rasheed et al., 2024). Longer residence times improve the material’s overall performance in environmental remediation (Kojić et al., 2022). Typical optimal conditions (500 °C–600 °C, 10 °C min^−1^, 2–4 h) yield biochars with surface areas of 150–250 m^2^ g^−1^ and superior adsorption capacities.

### 3.6 Residual mycelium and fungal biomass effect

SMS is a biologically transformed composite feedstock whose properties are fundamentally altered by fungal metabolism (Table 3). Residual mycelium, hyphal fragments, extracellular enzymes, and fungal-derived biopolymers (e.g., chitin, proteins, glucans) directly influence thermal decomposition pathways, pore development, surface chemistry, mineral speciation, and heteroatom incorporation during pyrolysis. These factors, in turn, govern key functional properties of SMS-derived biochars, including adsorption capacity, surface reactivity, nutrient release, alkalinity, and microbial interactions.

**TABLE 3:** Overview of mushroom species, SMS composition, and derived biochar properties.

Mushroom species *(Typical Substrate)*	SMS composition after cultivation	Biochar properties	Ref
*Pleurotus ostreatus* (Oyster; straw/sawdust)	Enriched in cellulose with residual lignin; fungal biomass adds N; moderate Ca from supplements	Alkaline; surface area ≈200 m^2^ g^−1^ ; Pb^2+^ adsorption ≈326 mg g^−1^ ; contains Ca and K	Zhang et al. (2022a)
*Lentinula edodes* (Shiitake; hardwood sawdust)	Extensive lignin degradation; enriched in CaCO_3_ and proteinaceous mycelium	Surface area up to 500 m^2^ g^−1^ ; Pb^2+^ adsorption ≈398 mg g^−1^ ; high Ca, K, P; strongly alkaline	Aiduang et al. (2025), Martín et al. (2023), Riquelme et al. (2018), Xiong et al. (2023), Zhang et al. (2024), Zhao et al. (2019)
*Agaricus bisporus* (Button; compost/manure/gypsum)	Nutrient-rich composted substrate; high N/P; residual CaSO_4_ and humic lignin	Ash ≈82%; pH ≈ 11.8; surface area peaks ≈101 m^2^ g^−1^ ; Pb^2+^ adsorption up to 564 mg g^−1^ (S-modified); strong metal sorption and slow-release fertilizer potential	Zhang et al. (2022b), Zhang et al. (2024)
*Ganoderma lucidum* (Reishi; sawdust)	Partial wood decomposition; CaCO_3_ residue; moderate fungal biomass	Surface area ≈425 m^2^ g^−1^ ; Pb^2+^ adsorption 142–263 mg g^−1^ ; high Ca; alkaline, stable, porous carbon structure	Martín et al. (2023), Zhang et al. (2024)
*Flammulina velutipes* (Enoki; sawdust + bran)	Lignin-rich SMS; elevated ash; enriched in P and metals from additives	Surface area ≈210 m^2^ g^−1^ at 400 °C; pH ≈ 11.6; ≈33% ash; P ≈ 1.2%; effective metal adsorption	Riquelme et al. (2018), Zhang et al. (2024)
*Tremella fuciformis* (Snow fungus; sawdust + host fungus)	Mixed fungal biomass; moderate lignin degradation; N-rich due to dual fungal protein content	N-enriched (≈2% N at low T); moderate porosity and adsorption; good nutrient release	Riquelme et al. (2018)
*Auricularia* spp. (Wood ear; sawdust)	Retains cellulose; Ca-rich from substrate; mycelial residues; moderate C:N ratio	Surface area up to 426 m^2^ g^−1^ ; Cd^2+^ ≈ 115 mg g^−1^ ; Cr^6+^ ≈ 118 mg g^−1^ ; Ca-rich; effective for heavy-metal adsorption and liming	Zhang et al. (2020)
*Volvariella volvacea* (Straw mushroom; paddy straw + manure)	Silica-rich (SiO_2_); partially composted straw; manure adds N, P, K	SiO_2_- and nutrient-rich; pH ≈ 9–11; improves soil structure and fertility	Zhang et al. (2022b)

#### 3.6.1 General mechanisms of fungal contribution

In addition to plant-based lignocellulosic matter, SMS also contains residual fungal biomass including mycelium, hyphae fragments, and extracellular metabolites left behind after mushroom cultivation (Riquelme et al., 2018). This biological component contributes significantly to the functionality and performance of the resulting biochar. Specifically, the fungal residues contribute to the development of pore structure, influence microbial colonization potential, and affect surface reactivity, thereby adding another layer of complexity to the physicochemical properties of biochar produced from SMS.

During pyrolysis, fungal biomass, rich in chitin, proteins, and polysaccharides, decomposes differently than plant-derived cellulose and lignin (Xiong et al., 2023). These differences result in distinct gas evolution and thermal breakdown, which facilitate the formation of micropores and mesopores, thereby increasing the surface area and adsorptive capacity of the resulting biochar. Additionally, the presence of fungal residues contributes to the incorporation of nitrogen- and oxygen-containing groups on the biochar surface. These groups enhance the chemical reactivity of the material and improve its affinity for a variety of pollutants such as heavy metals, dyes, and pharmaceuticals. These functional moieties promote microbial colonization, further increasing the biochar’s potential for use in soil and environmental applications.

The specific fungal species used in mushroom production further impact the final biochar properties. Different mushroom species produce distinct extracellular enzymes and have varied preferences for nutrient uptake, which affects what remains in the SMS.

#### 3.6.2 Species-specific case studies

**FIGURE 4:**
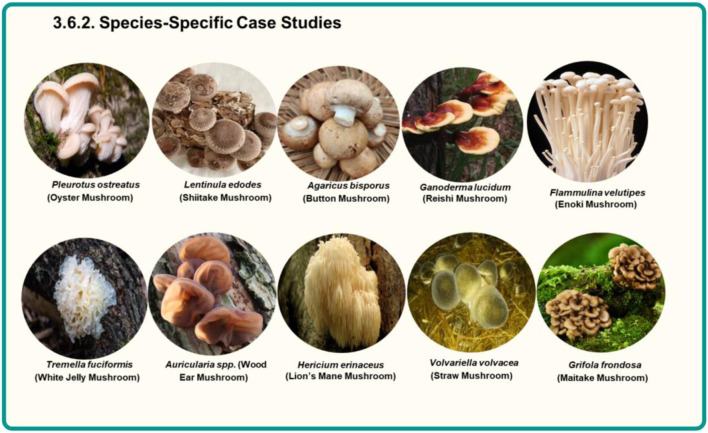
Representative mushroom species used in species-specific case studies of spent mushroom substrate (SMS). Different fungal metabolisms associated with genera such as *Pleurotus*, *Lentinula*, *Agaricus*, *Ganoderma*, *Flammulina*, *Tremella*, *Auricularia*, *Hericium*, *Volvariella*, and *Grifola* selectively modify lignocellulosic components of the growth substrate, altering mineral composition, nitrogen incorporation, and functional group chemistry. These biological preconditioning effects contribute to the variability observed in downstream thermochemical conversion performance and biochar properties.

The species-specific case studies presented in this section are quantitative structure–property analyses demonstrating how different fungal metabolisms precondition the substrate prior to thermochemical conversion. As shown for *Pleurotus*, *Lentinula*, *Agaricus*, *Ganoderma*, *Flammulina*, *Tremella*, *Auricularia*, and other genera (Figure 4), fungal growth selectively removes or modifies lignocellulosic fractions, concentrates specific minerals, and introduces nitrogen- and oxygen-containing functional groups. These feedstock-level differences explain the wide variability observed in biochar yield, ash content, surface area, pH, and pollutant removal performance across studies.

##### 3.6.2.1 *Pleurotus ostreatus* (oyster mushroom)

*Pleurotus ostreatus* is a fast-growing primary decomposer often cultivated on wheat straw or sawdust. As a white-rot fungus, it produces enzymes that break down all components of lignocellulose – though in practice it consumes hemicellulose and lignin more extensively than cellulose (Martín et al., 2023). Reported mass losses during *Pleurotus* cultivation indicate that 57%–77% of hemicelluloses and 61%–75% of lignin are degraded, against only ∼26–46% of the cellulose (Martín et al., 2023). This means *Pleurotus* SMS tends to be enriched in cellulose relative to the starting material and has a higher proportion of easily charred carbon. The SMS also contains substantial fungal biomass (oyster mushroom mycelium), which is protein-rich and adds nitrogen to the substrate. Any supplements (e.g., wheat bran or lime) contribute to residual minerals. As a result, oyster mushroom SMS typically has a moderate C:N ratio with abundant organic matter and some mineral content.

When converted to biochar, Pleurotus SMS yields a highly porous, functionalized carbon. Studies show oyster mushroom biochars are alkaline and rich in surface functional groups (due to the protein/chitin content) that aid adsorption (Ravlikovsky et al., 2024). They also exhibit high surface area – on the order of 150–250 m^2^/g under moderate pyrolysis conditions (e.g., 400 °C–500 °C) (Zhao et al., 2019). One notable feature is the ability to adsorb heavy metals, especially lead (Pb). Biochar made from *P. ostreatus* spent substrate achieved a Pb^2+^ adsorption capacity of ∼326 mg/g in tests, which is among the highest for unmodified biomass chars (Ravlikovsky et al., 2024). This performance is attributed to its porous structure and nutrient-derived minerals that can complex with metals (Ravlikovsky et al., 2024). The char also contains base cations like Ca^2+^, K^+^ from the substrate, giving it a liming effect and cation exchange capacity. Overall, *P. ostreatus* SMS biochar combines high porosity and adsorption capacity with a balanced nutrient profile, making it effective as a soil amendment and pollutant sorbent.

##### 3.6.2.2 *Lentinula edodes* (shiitake mushroom)

Shiitake mushrooms (*L. edodes*) are grown on hardwood sawdust blocks (or logs) supplemented with nutrients (e.g., rice bran) and usually buffered with calcium carbonate. *Lentinula* is another white-rot fungus; it degrades lignin and hemicellulose into wood, though it works somewhat slower than *Pleurotus*. When this spent substrate is pyrolyzed, it produces a calcium-rich biochar with remarkable properties. Due to the residual CaCO_3_, shiitake biochar can contain a high proportion of calcite (CaCO_3_) and other Ca compounds (Zhang H. et al., 2022). This yields an alkaline char (pH ∼10–11) that can strongly neutralize acids. Additionally, the presence of Ca (and Mg) leads to the formation of mineral carbonates and oxides that enhance heavy metal removal via precipitation/exchange. For example, *L. edodes* SMS-derived biochar has shown an exceptional capacity for Pb^2+^ adsorption – up to ∼398 mg/g in batch experiments. This is attributed to mechanisms like Pb^++^ exchanging with Ca^++^ and forming stable PbCO_3_ on the biochar surface (Zhang H. et al., 2022). Additionally, biochar from *Lentinus edodes* (shiitake) SMS exhibits selective adsorption of tetracycline and Fe ions, (Wang J. et al., 2024), due to presence of nitrogen-rich extracellular compounds, including amino acids and enzymes. These compounds contribute to the formation of nitrogen-doped functional groups on the biochar surface during pyrolysis, which enhance electrostatic interactions and complexation with metal ions and polar organic molecules, such as tetracycline.

The surface area and porosity of shiitake biochars are also impressive. Owing to the thorough breakdown of the substrate (and perhaps the templating effect of minerals), shiitake SMS biochar can achieve very high BET surface areas. One study reported ∼312 m^2^/g at 800 °C for shiitake compost char, (Chen et al., 2022) and another found over 500 m^2^/g at 750 °C when comparing shiitake vs. reishi chars (Zhang H. et al., 2022). In general, it consistently outperformed other species in pore development (e.g., at every temperature, *Lentinula* char had larger surface area than *Ganoderma* char) (Chen et al., 2022). The nutrient content of the char is noteworthy as well – shiitake biochar retains significant K (up to ∼2% K) and P, derived from the supplemented bran (Zhao et al., 2019). These nutrients, along with Ca, make it a slow-release fertilizer when applied to soil (Aiduang et al., 2025). In summary, *L. edodes* spent substrate yields biochar that is highly porous, strongly alkaline, and laden with nutrients, excellent for immobilizing contaminants and improving soil fertility.

##### 3.6.2.3 *Agaricus bisporus* (button mushroom)

The button mushroom is unique among those discussed in that it is a secondary decomposer, grown on a composted substrate. *A. bisporus* cultivation uses a manure-enriched straw compost, typically with added gypsum (CaSO_4_) and a top layer of peat soil casing. By the end of cropping, spent mushroom compost (SMC) is a well-stabilized, humus-like material. It is very rich in organic nutrients (nitrogen, phosphorus) from manure, and in minerals like calcium (from gypsum) (Zhang et al., 2020). Much of the easily degradable cellulose and sugars were consumed or decomposed during composting and mushroom growth. The remaining substrate is high in lignin-like substances and microbial biomass from the composting phase.

Pyrolyzing *Agaricus* SMC produces a mineral-rich biochar with some extreme characteristics. Because the feedstock is loaded with inorganic matter (soil, ash, gypsum), the char yield is very high–one study reported a yield of ∼64.6% at 750 °C, (Zhang et al., 2020), *versus* typical yields of 20%–30% for pure lignocellulosics. Correspondingly, the ash content of the char was enormous (82.1% at 750 °C) (Zhang et al., 2020). This ash consists of Ca, Mg, P, K, Si and other mineral elements that were not volatilized. The char’s pH is strongly alkaline (raw SMC pH ∼6.9, but char pH rose to 11.8 at 750 °C) due to the conversion of gypsum and other salts into oxides/carbonates (e.g., CaCO_3_, CaO) during pyrolysis. The high mineral content also means high electrical conductivity and cation exchange capacity, which can benefit soils.

In terms of surface area, SMC-derived char tends to develop porosity up to an optimal point, then decline if excessive ash blocks the pores. For example, one study noted surface area increased from ∼36 m^2^/g at 350 °C to ∼101 m^2^/g at 650 °C, but then *decreased* to ∼37 m^2^/g at 750 °C as ash accumulation clogged pores (Zhang et al., 2020). Thus, SMC char might not rival wood-char in surface area if pyrolyzed at very high temperature without pretreatment. However, it more than makes up for this with chemical functionality: Agaricus SMC biochar excels at heavy metal adsorption through precipitation and ion exchange. Research has shown that at 750 °C, spent Agaricus substrate char can remove Cu^2+^ (∼68.1 mg/g), Zn^2+^ (∼55.2 mg/g), and Cd^2+^ (∼64.8 mg/g) from solution, outperforming many conventional biochars (Aiduang et al., 2025). These values were higher than those for chars made from pure straw or wood (Zhang et al., 2020). The dominant removal mechanism at high temperature was found to be mineral precipitation (e.g., forming insoluble carbonates or hydroxides with Ca/Mg) (Dickson and Cornish, 2025). Indeed, the mineral-rich char provides so many basic sites that it can neutralize acidic solutions and cause metals to precipitate as carbonates/phosphates on its surface. In some cases, researchers have further modified SMC biochar (e.g., magnetization, sulfur impregnation) to achieve extraordinarily high adsorption capacities – one report cited >500 mg/g for Pb^2+^ and ∼332 mg/g for Zn^2+^, (Aiduang et al., 2025), which underscores the potential of this feedstock. Nutrient-wise, Agaricus-char retains significant phosphorus (often 1%–3% P) and nitrogen (0.5%–1% N), (Aiduang et al., 2025) making it a nutrient source. In summary, *A. bisporus* spent compost yields biochar with extremely high ash and nutrient content, strongly alkaline and effective in binding heavy metals, though with somewhat lower surface area due to pore blockage by minerals.

##### 3.6.2.4 *Ganoderma lucidum* (reishi mushroom)

*Ganoderma lucidum*, a medicinal mushroom, is typically cultivated on sterilized hardwood sawdust blocks (often with minimal supplements compared to edible mushrooms). It is a white-rot wood decomposer as well, so its SMS is qualitatively similar to that of shiitake: partially degraded wood fibers, rich in cellulose and with much lignin removed. However, *Ganoderma* grows slowly and may not consume as high a fraction of the substrate by the end of the growing cycle. The spent substrate still contains a fair amount of structural wood components alongside the Ganoderma mycelium (which is less protein-rich than oyster or shiitake). Supplements like wheat bran (if used) add some nutrients, and typically calcium carbonate is added as a pH buffer in the blocks (so Ca remains in SMS).

Upon pyrolysis, *Ganoderma* SMS produces a stable, carbon-dense biochar with properties akin to other hardwood-based biochars but slightly moderated by a bit lower nutrient loading than shiitake. A recent comparative study of biochars from G. lucidum vs. L. edodes spent substrates found that both were “calcium-rich” chars dominated by Ca minerals, but the shiitake char had even more ash and Ca than the reishi char (Zhang H. et al., 2022). At 750 °C, Ganoderma char had a BET surface area of ∼426 m^2^/g, whereas shiitake char reached ∼503 m^2^/g (Zhang H. et al., 2022). This indicates that while Ganoderma char is highly porous, it may have slightly fewer pores or more collapsed structure compared to shiitake char (possibly due to differences in how the fungi modify the substrate’s microstructure). Nonetheless, a surface area in the 400–500 m^2^/g range is excellent and implies strong adsorption potential.

Heavy metal adsorption tests reflect a good but variable performance for Ganoderma char. The MDPI 2025 review summarizes that *G. lucidum*-derived biochar showed cadmium adsorption ranging from ∼23.8 up to 75.8 mg/g, and lead from ∼141.6 up to 262.8 mg/g (Aiduang et al., 2025). This wide range suggests that different preparation conditions or post-treatments of the char can greatly affect its adsorption efficiency (e.g., activation, particle size, pyrolysis temperature all matter). In general, unmodified reishi char should effectively immobilize heavy metals due to its abundant basic carbonates (from Ca, Mg) and functional groups. It is also likely effective for organic pollutant adsorption given its high surface area and aromatic carbon structure (Zhang H. et al., 2022).

Nutrient-wise, Ganoderma SMS char contains substantial calcium (not quite as high as shiitake, but still often the dominant mineral), plus magnesium, potassium, and phosphorus derived from the wood and any bran supplement (Zhang H. et al., 2022). These give it value as a soil additive to supply secondary nutrients and raise soil pH. Furthermore, Ganoderma char’s carbon is quite recalcitrant (coming from lignin-rich wood), contributing to long-term soil carbon sequestration and stability (Aiduang et al., 2025). In summary, *G. lucidum* spent substrate yields high-quality biochar that is highly porous and alkaline, with strong but slightly variable adsorption capabilities and a mineral profile that improves soil fertility.

##### 3.6.2.5 *Flammulina velutipes* (Enoki mushroom)

Enoki mushrooms are cultivated on substrates like sawdust, corn cobs, or other agrowaste with supplements, and Enoki is a fast-growing fungus. It is considered a primary decomposer as well, capable of decomposing lignocellulose though perhaps not as completely as longer-cycle mushrooms. By the end of the short cultivation cycle for *F. velutipes*, the SMS still contains a significant amount of lignocellulosic material (though somewhat softened), along with the threadlike mycelium of the fungus. One study (Zhao et al., 2019) compared *F. velutipes* spent substrate to others and found that the Enoki SMS had the highest residual ash content after pyrolysis, suggesting its substrate or growth left more inorganic residue.

When Enoki SMS is converted to biochar, one standout characteristic is its high surface area at relatively low pyrolysis temperatures. Zhao et al. (2019) reported that among biochars made at 300 °C–700 °C from three fungi, the *F. velutipes* char had the largest surface area–with Fv-400 °C reaching ∼210.6 m^2^/g, higher than chars from Tremella or Shiitake at the same temperature. This indicates Enoki SMS might develop microporosity readily, perhaps because of the structure of the partially decomposed corn cob/cellulose it leaves behind. However, as temperature increased to 700 °C, Enoki char also accumulated the most ash (33.4%) (Zhao et al., 2019) and had the highest pH (∼11.6), implying a lot of inorganic content (possibly silica from plant material, calcium from supplements) remained and contributed to alkalinity. The high ash at 700 °C can sometimes clog pores, but even so, Enoki char’s surface area and pore volume remain substantial. It also had the highest phosphorus content of the chars examined (P ≈ 12 g/kg at 700 °C), (Zhao et al., 2019) likely reflecting bran or corn cob minerals that were concentrated in the ash.

In terms of adsorption performance, *Flammulina* SMS char is effective across a range of pollutants. Although not singled out in heavy-metal capacity comparisons in the review, it can be inferred to perform similarly well. The combination of high microporosity and abundant surface functional groups (Zhao et al., 2019) noted C–O groups dominated its surface spectra) helps Enoki char adsorb heavy metals and organics (Zhao et al., 2019). The char also had the highest measured CEC (cation exchange capacity) in one case (32.3 cmol/kg for Fv-600), which demonstrates its ability to retain cationic nutrients and contaminants. Nutrient-wise, beyond P, Enoki char carries moderate levels of K, Ca, Mg (depending on substrate composition) and is overall beneficial for soil amendment. Its high alkalinity can correct acidic soils while its porosity improves water retention (Aiduang et al., 2025). In summary, *F. velutipes* SMS yields biochar with notably high surface area and pore volume, high ash/alkalinity at higher pyrolysis, and solid adsorption and nutrient-retention capabilities–a well-rounded soil and environmental material.

##### 3.6.2.6 *Tremella fuciformis* (white jelly mushroom)

*Tremella fuciformis* is an interesting case: it’s a parasitic fungus that in cultivation is often grown in tandem with a host fungus (e.g., a species of *Annulus* or *Lentinus* that parasitizes). Cultivators usually inoculate sawdust with a host, then with *Tremella*. This means the spent substrate may contain remnants of two fungi’s activity. The host fungus begins breaking down the sawdust, and Tremella feeds off the host mycelium. By harvest, the SMS composition includes partially decomposed wood (the host’s work) and a large amount of fungal biomass (both host and Tremella). The nitrogen content of Tremella SMS can be relatively high because Tremella is rich in polysaccharides and also because the host fungus is present (effectively boosting microbial protein content). The substrate has a good fraction of cellulose (since the host and Tremella together will not fully consume it in the given time) and some lignin.

Upon pyrolysis, *Tremella* spent substrate gives a biochar that is distinguished by its higher nitrogen content and volatile matter at low temperatures. Zhao et al. (2019) found that Tremella-char at 300 °C retained ∼2.07% N (highest among the fungi tested) (Zhao et al., 2019) and had a very high volatile matter (79.6% VM at 300 °C). At moderate pyrolysis, Tremella char still contains many organic compounds (likely due to the high fungal-derived content). At higher pyrolysis temperatures those volatiles crack off, and the char becomes more carbon-rich. In Zhao’s FTIR analysis, Tremella char showed a variety of functional groups, with C–O stretching being common (similar to others) (Zhao et al., 2019). Tremella SMS had no extraordinary mineral addition beyond what the host’s substrate had.

In practical terms, Tremella SMS biochar is a good multi-purpose biochar. It is nutrient-rich – containing more N than typical biochars, plus moderate P, K from the sawdust/bran – which can act as a fertilizer. Its adsorption capacity is not highlighted as exceptional; still, with N/O-functional groups, it can bind metals via surface complexation. It’s also effective for organic pollutant adsorption given its microporosity and polar surface sites. Specific performance data are rare for *Tremella* char.

##### 3.6.2.7 Auricularia spp (wood ear mushroom)

Auricularia (e.g., *A. auricula-judae*, the black wood ear fungus) is cultivated on wood-based substrates similar to shiitake or maitake – usually sawdust with some supplements, packed in bags. It’s also a white-rot fungus, though it tends to have a gelatinous fruiting body and might not degrade the wood as aggressively as, say, *Pleurotus*. The SMS from *Auricularia* cultivation is composed of partially decayed sawdust with a fair amount of residual cellulose and lignin (depending on cultivation time and conditions). Nutrient additives (bran, gypsum) leave mineral traces; indeed, farmers often add lime/gypsum, so Ca content in Auricularia SMS can be notable. There may also be a unique aspect: some studies indicate Auricularia can produce organic acids, possibly affecting the substrate’s chemical makeup (e.g., partial delignification via acid hydrolysis). In any case, the spent substrate is lignocellulosic and nutrient-enriched by fungal biomass.

Biochar derived from *Auricularia* SMS has attracted research interest for pollutant removal. A key finding is that *Auricularia*-based biochar can be exceptionally effective for certain heavy metals. For instance, one study reported that biochar from *A. auricula* substrate had the highest efficiency for Cr(VI) and Cd(II) removal among several mushroom chars – ∼118 mg/g for Cr and ∼114.6 mg/g for Cd (Aiduang et al., 2025). These high values were likely achieved by chemically modifying the char (the study used CS_2_ to sulfur-modify the biochar, (Li et al., 2018) introducing sulfur functional groups that strongly bind soft metal ions like Cd). Even without such modification, *Auricularia*-char benefits from inherent surface functionality and mineral content. It typically contains a lot of Ca (like shiitake char does) which helps precipitate metals, and its surface may have carboxyl/hydroxyl groups from partially charred polysaccharides that can coordinate metal ions (Aiduang et al., 2025). The porous structure of Auricularia char is well-developed: in one study where shiitake vs. black fungus chars were made at 800 °C, the Auricularia (termed “black fungus”) char had ∼281 m^2^/g surface area, (Chen et al., 2022) only slightly less than the shiitake’s 312 m^2^/g. At 750 °C, another study found *Auricularia*-char reaching ∼426 m^2^/g (comparable to Ganoderma in that study) (Zhang H. et al., 2022). These numbers show that, under high-temperature pyrolysis, *Auricularia* SMS yields a highly porous carbon matrix.

Another interesting trait is that *Auricularia* substrate often includes calcium-rich additives (like ground limestone), which means the resulting char can be considered a form of “calcium-rich biochar.” In fact, separate research on Auricularia SMS char has looked at dye adsorption and found it very effective when activated, indicating a broad spectrum of use (dyes, heavy metals, etc.) (Zhang H. et al., 2022)

In summary, *Auricularia* spent substrate produces biochar that is highly porous and alkaline, with excellent adsorption capacity especially when enhanced with modifiers. It carries a suite of nutrients/minerals (Ca, K, Mg, etc.) similar to other wood ear feedstocks, making it beneficial as a soil amendment. Its performance in removing pollutants like Cr^6+^, Cd^2+^, Pb^2+^ is among the top tier of SMS biochars (Ravlikovsky et al., 2024) reported.

##### 3.6.2.8 *Hericium erinaceus* (Lion’s mane mushroom)

*Hericium erinaceus* is cultivated on hardwood sawdust blocks with nutrients (commonly rice or wheat bran) and sometimes additives like soybean meal or gypsum. It is a slower-growing fungus (compared to *Pleurotus*), but it does colonize and decay the substrate, primarily consuming cellulose and hemicellulose. By the end of a *Hericium* crop, the SMS is a compacted sawdust block that has been partially degraded – typically softer and lighter in color than when started, indicating breakdown of some lignin as well. The mycelial biomass in Hericium SMS is also notable; Lion’s Mane mycelium can be thick and fibrous, adding some protein and glucans to the residue. Additionally, any gypsum added (commonly ∼5% CaSO_4_ is used in gourmet mushroom substrates) remains as CaSO_4_ or converts to CaCO_3_ during drying. When *Hericium* SMS is pyrolyzed, it yields a biochar with well-balanced properties, however, there isn’t a lot of published data specifically on *Hericium* biochar.

##### 3.6.2.9 *Volvariella volvacea* (straw mushroom)

*Volvariella volvacea* is commonly cultivated on paddy straw beds or heaps (often outdoors or in simple structures). The cultivation process usually involves a short high-temperature fermentation of straw (sometimes with added manure or fertilizers) to create a suitable substrate. The fungus then grows and fruits on this semi-composted straw. The spent substrate from *Volvariella* is essentially spent straw compost, rich in microbial metabolites from the fermentation, and possibly with residual manure. Key features of this SMS: it’s high in silica (rice straw contains a lot of SiO_2_ in its epidermis), relatively fibrous (since not all straw is consumed), and contains some nutrient enrichment (N, P, K from any added manure or from straw’s own content). *Volvariella* mycelium itself is not as nutrient-dense as *Agaricus* or *Pleurotus*, but it still adds some protein.

Converting straw mushroom SMC into biochar yields a product somewhat distinct from wood-based biochars due to the high silica content. Silica is very thermally stable and ends up in the char ash as inert SiO_2_ particles. A “silica-rich biochar” can be advantageous for certain uses (such as reinforcing soils or adsorbing organic compounds, since silica can add surface area and polarity). If manure was used, that adds minerals too (notably phosphorus and calcium).

In terms of structure, straw-derived biochars often have a tubular, porous structure (coming from the vascular bundles in straw). This usually translates to decent surface area with actual values depending on conditions. The straw mushroom char’s pores are a mix of meso- and micropores, although there is no specific study on *Volvariella* SMS char adsorption. If we compare to generic rice-straw char or other agri-waste chars, those have shown good heavy metal adsorption (for example, rice straw char might remove ∼20–50 mg/g of Cu or Cd under typical conditions) and excellent performance for dyes and ammonia, etc., especially if slightly activated (Zhang et al., 2020). If manure was present, the char’s nitrogen content could be a bit higher than plain straw char, and the phosphorus content would certainly be elevated which would make *Volvariella* char a valuable slow-release fertilizer.

##### 3.6.2.10 *Grifola frondosa* (maitake mushroom)

*Maitake* (Hen-of-the-woods) is cultivated on sterilized hardwood sawdust blocks with bran supplements, similar to shiitake. It’s a slow-growing polypore that eventually produces large cluster fruits. During its lengthy incubation and fruiting period, *Grifola frondosa* mycelium extensively colonizes the sawdust and degrades a significant portion of the wood’s polysaccharides. Maitake is a white-rot fungus, so it can break down lignin as well, although anecdotal reports suggest it may not be as aggressive in lignin degradation as shiitake – it tends to prefer cellulose and hemicellulose first. By the end of a maitake crop, the block is somewhat lightweight and partially degraded, but typically some lignin-rich material remains (giving the spent substrate a somewhat brown color and fibrous texture). The spent substrate is also packed with *Grifola* mycelium (which is edible to some extent and sometimes used as animal feed supplement). Nutrient supplements like bran contribute extra nitrogen, phosphorus, and especially potassium (since wood itself is low in K, bran supplies it). Usually, a bit of gypsum or lime is used in Maitake substrate as well, so Ca is present.

When converting *G. frondosa* SMS to biochar, we expect a high-carbon char due to the relatively longer fungal growth and higher carbon retention in the substrate. Maitake SMS might not have as much ash as shiitake or auricularia (depending on how much supplement was used), but it still will have more than plain wood because of the bran and lime. The biochar is therefore likely alkaline and nutrient enriched. It will contain calcium carbonate (from the lime/gypsum, which in pyrolysis forms CaO then captures CO_2_ to become CaCO_3_ upon cooling) and potassium compounds (K_2_CO_3_ from the ashes of bran/wood). These confer a high pH and the ability to supply K and Ca to soil. The phosphorus from bran will largely remain in the char as stable calcium phosphate or similar, making it a slow-release P source.

The surface area measurements are not published. The presence of slightly more residual lignin in maitake SMS might increase char yield and favor the formation of a robust pore structure (lignin chars tend to form more aromatic, solid char). As for adsorption capacity, maitake char has not been singled out in comparative studies.

#### 3.6.3 Organic and moisture content

The high organic and moisture content of SMS strongly affects the pyrolysis process and biochar yield. Moisture content, which can exceed 60%, reduces pyrolysis efficiency and requires energy-intensive drying. However, once optimized, pyrolysis of SMS with balanced organic content results in functionalized surfaces that promote pollutant adsorption and soil microbial activity. Tailoring pyrolysis temperature and residence time is essential to maximizing carbon retention and surface chemistry development (Alves et al., 2021).

#### 3.6.4 Applications for spent mushroom substrate (SMS)-derived bio-chars

Spent mushroom substrate (SMS) is increasingly being recognized as a valuable resource for environmental remediation and pollutant management due to its ability to be transformed into biochar with multifunctional applications. SMS-derived biochar exhibits, as mentioned before, remarkable properties such as high porosity, large surface area, and an abundance of functional groups. This makes it suitable for addressing critical issues in heavy metal removal, organic pollutant adsorption, and soil amendment.

The ability of SMS-derived materials to remove heavy metals from aqueous solutions has been extensively studied, with promising results. For example, biochar prepared using tubular furnaces and pyrolyzed at 400 °C–800 °C has been shown to adsorb lead and cadmium ions with capacities reaching 90.58 mg/g and 25.348 mg/g, respectively (Xu et al., 2024b). Similarly, biochar from wheat bran and sawdust, pyrolyzed at 500 °C, exhibits adsorption efficiencies of 52.6–65.6 mg/g for copper ions and significant removal of zinc ions (Fan et al., 2024). These findings underscore the importance of optimizing the substrate composition and pyrolysis conditions to achieve high metal-binding efficacy.

In addition to heavy metal adsorption, SMS-derived biochar has demonstrated outstanding capabilities in removing organic pollutants such as dyes and pharmaceuticals from wastewater. Studies reveal that biochar from Ganoderma lucidum SMS adsorbs dyes like malachite green and safranin T with exceptional capacities of over 9,388 mg/g and 3,871 mg/g, respectively (Corral-Bobadilla et al., 2019). Similarly, tetracycline removal using iron-modified biochar derived from Lentinus edodes exhibits adsorption capacities ranging from 9.63 mg/g to 37.95 mg/g, depending on the iron valence used in the modification process (Wang Y. et al., 2024). Such applications are critical for mitigating pollution in water systems.

Research on SMS-derived biochar highlights its remarkable versatility as both a carbon-rich adsorbent and a nutrient-releasing soil amendment. The interplay among substrate composition, fungal species, and pyrolysis parameters governs the physicochemical outcomes, from mineral-rich, alkaline chars suited for heavy-metal immobilization to nitrogen-doped, high-surface-area carbons optimized for dye and pharmaceutical adsorption. Residual fungal biomass imparts heteroatom functionality and microporosity, while mineral additives such as Ca, K, and P enhance ion-exchange capacity and buffering potential. These findings demonstrate that SMS is not a waste by-product but a strategic feedstock for producing engineered biochars tailored to specific environmental or agricultural applications. Future work should prioritize standardized processing protocols, activation strategies, and life-cycle assessments to translate SMS-based biochar production from laboratory to scalable industrial practice.

## 4 Biofuels from SMS

Biofuel refers to renewable fuel produced from organic matter or biomass, serving as an alternative to fossil fuels. These fuels can be in solid, liquid, or gaseous forms, such as bioethanol, biodiesel, or biogas, and are considered more environmentally friendly due to their potential to reduce greenhouse gas emissions and their renewable nature.

Biofuels derived from spent mushroom substrates (SMS) offer a sustainable solution for renewable energy production. SMS consists of lignocellulosic biomass leftover from mushroom cultivation, which would otherwise be discarded as waste. Given its organic composition, SMS is an ideal feedstock for biofuel conversion through various biochemical and thermo-chemical processes.

The production of biofuels from SMS involves enzymatic saccharification, anaerobic digestion, pyrolysis, and gasification. These processes result in ethanol, methane, syngas, bio-oil, and solid fuel pellets (Yin Y. et al., 2025) demonstrated the potential of SMS from Shiitake mushrooms, yielding 84.40% reducing sugars, supporting ethanol production. Similarly, anaerobic digestion of SMS combined with brewers’ spent grain enhanced methane generation (Ganuza et al., 2023). Thermo-chemical processing methods such as pyrolysis have been successfully used to produce bio-oil from SMS mixed with oil shale semi-coke (Jiang et al., 2017).

Pretreatment strategies significantly affect SMS biofuel production efficiency (Koido et al., 2021) 93explored steam co-gasification of SMS blended with Japanese cedar, producing high-yield syngas (Kumar et al., 2021) used electrokinetic-assisted anaerobic digestion to optimize biogas yield, highlighting advancements in SMS valorization techniques. Additionally, SMS has been densified into bio-coke for combustion applications (Koesoemadinata et al., 2023). Table 4 summarizes reported studies on SMS conversion under various pretreatment, enzymatic, and thermochemical conditions, grouped according to their respective biofuel products. The principal biochemical and thermochemical pathways for converting SMS into biofuels are summarized schematically in Figure 5.

**TABLE 4:** Substrate materials used for mushroom cultivation and subsequent biofuel production: fungal species, pretreatment methods, enzymatic hydrolysis conditions, and biofuel produced.

#	Substrate	Species	Pre-treatment condition	Enzyme	Saccharification condition	Total reducing sugar (%)	Biofuel	Ref
(a) Biochemical Conversion – Ethanol, Biobutanol, Hydrogen								
1	Hardwood chips, wheat straw, wheat bran and calcium carbonate	*L. edodes*	Biological pretreatment using white-rot fungi	Cellic CTec2 (blend of cellulases, β-glucosidases,hemicellulases)	72 h, 50 °C, pH 5.2, 100 CMCase units/g biomass	84.40	Ethanol	Yin et al. (2025a)
2	Hardwood chips, wheat straw, wheat bran and calcium carbonate	*Ganoderma lucidum*	Biological pretreatment using white-rot fungi	Cellic CTec2	72 h, 50 °C, pH 5.2, 100 CMCase units/g biomass	33.50	Ethanol	Yin et al. (2025a)
3	Hardwood chips, wheat straw, wheat bran and calcium carbonate	*Pleurotus ostreatus*	Biological pretreatment using white-rot fungi	Cellic CTec2	72 h, 50 °C, pH 5.2, 100 CMCase units/g biomass	Poor saccharification	Not specified	Yin et al. (2025a)
4	Not specified	*Pleurotus ostreatus*	Hybrid pretreatment (Microwave, Alkali, Ultrasound)	Cellulase enzyme (*Trichoderma reesei* ATCC 26921)	60 h, 45 °C, pH 4.8, 30 FPU/g biomass	52.15	Biobutanol	Suresh et al. (2024)
5	Not specified	Not specified	Biological pretreatment using *Clostridium thermocellum* 27,405 + recombinant β-glucosidases	β-Glucosidase (wild-type and fusion versions)	Fermentation at 55 °C, 150 rpm for 168 h	50.6	Hydrogen	Lin et al. (2015)
(b) Anaerobic Digestion – Methane and Biogas								
6	Brewers’ spent grain (BSG)	*Pleurotus pulmonarius*	Sterilization (autoclaving at 121 °C for 2.5 h)	Not specified	Used as substrate for mushroom cultivation, then for biogas generation	Not specified	Methane	Ganuza et al. (2023)
7	Not specified	*Pleurotus pulmonarius*	Mushroom cultivation (BSG + sawdust substrate, colonized for 30 days)	Not specified	Used in anaerobic digestion in mesophilic conditions (35 °C)	Not specified	Methane	Ganuza et al. (2023)
8	Rice Straw (RS)	ND	Integrated alkaline-solid/liquid separation-thermal multiple-step pretreatment (AK-SL-TP)	Cellic CTec3	50 °C, pH 5, 24 h	55.97	Methane	Wang et al. (2025)
9	Rice Husk (RH)	ND	Integrated alkaline-solid/liquid separation-thermal multiple-step pretreatment (AK-SL-TP)	Cellic CTec3	50 °C, pH 5, 24 h	27.31	Methane	Wang et al. (2025)
10	Not specified	*Agaricus bisporus*	Electrokinetic-assisted anaerobic digestion	Not specified	34.52 °C, 1.61 V, 59.61% Sugar Mill Wastewater (SMW)	Not specified	Biogas	Sudhakar et al. (2021)
11	Livestock manure	*Morchella esculenta*	Co-digestion with chicken, dairy, or pig manure under varying total solids (TS) content and manure ratios	ND	Optimal methane yield at 15% TS, SMS-to-manure ratio of 1:2, with adjusted pH = 7.0 and anaerobic conditions maintained at 35 °C	ND	Methane	Gao et al. (2021)
12	ND	*Flammulina velutipes (SFv), Pleurotus eryngii* var. *tuoliensis (SPt), Pleurotus eryngii (SPe)*	Co-digestion with dairy manure (DM) at different ratios (1:1, 3:1, 1:3)	ND	Anaerobic digestion, 49-day batch tests at mesophilic conditions (36 °C ± 2 °C)	ND	Methane	Luo et al. (2018)
13	Waste Paper	*Pleurotus florida*	Briquetting under high pressure in a screw-type machine	ND	Anaerobic digestion at 37° C for 60 days	ND	Methane	Selvan and P (2017)
(c) Thermochemical Conversion – Bio-Oil, Syngas, Bio-Coke, Solid Fuels, and Activated Carbon								
14	Spent Mushroom Substrate (SMS)	Not specified	Thermo-chemical pyrolysis with CO_2_ as reactive gas	Ni-based catalyst	≥500 °C in CO_2_ environment	Not specified	Syngas (H_2_ + CO)	Lee et al. (2024b)
15	Not specified	*Pleurotus ostreatus*	Densification using high pressure (21.7 MPa) and moderate temperature (130 °C–190 °C)	Not specified	Compressive testing, TGA analysis for combustion	Not specified	Bio-Coke	Koesoemadinata et al. (2023)
16	Not specified	*Pleurotus* sp.	Densified using a high-pressure (21.7 MPa) and moderate temperatures (130 °C–190 °C)	Not specified	Combustion-related mechanical durability tested	Not specified	Bio-Coke	Aniza et al. (2022)
17	Not specified	*Pleurotus* sp.	Densified using a high-pressure (21.7 MPa) and moderate temperatures (130 °C–190 °C)	Not specified	Combustion-related mechanical durability tested	Not specified	Bio-Coke	Devi et al. (2022)
18	Not specified	ND	Thermochemical torrefaction and pyrolysis via microwave irradiation with MgO catalyst and ferric ammonium sulfate as magnetic agent	Not specified	30 min at 900 W (power) and 355 µm particle size	Not specified	Bio-oil	Kumar et al. (2021)
19	Paddy Straw	*Pleurotus ostreatus*	Thermomechanical pelletization under high pressure (200 MPa) and 125 °C	Not specified	Combustion trials at ∼315 °C–338 °C ignition temperature	Not specified	Solid biofuel pellets	Alves et al. (2021)
20	Compost with Soil Casing	*Agaricus subrufescens*	Similar pelletization process as above	Not specified	Combustion stability and emissions tested extensively	Not specified	Solid biofuel pellets	Alves et al. (2021)
21	Not specified	*Lentinula edodes*	Steam co-gasification blended with Erianthus or Japanese cedar	Not specified	Operated at T ≥ 800 °C–900 °C with steam-to-carbon (S/C) ratio of 1	Not specified	Syngas (H_2_ + CO)	Koido et al. (2021)
22	Not specified	*Agaricus bisporus*	Dual processing: Carbonization + Activation, incorporating flue gas	Not specified	Gasifier set at ≥750 °C	Not specified	Activated Carbon	Liu et al. (2021)
23	Briquettes made of sugarcane bagasse	*Pleurotus ostreatus* var. *Florida*	Briquetting at varied pressures (45 MPa and moisture content between 60% and 80%) followed by drying	ND	Fuel briquettes tested for heat density and combustion duration under controlled environment	ND	Fuel briquettes	Leong et al. (2022)
24	ND	*Pleurotus ostreatus*	Combined thermochemical and microwave-assisted pre-treatment. Optimized High-purification-step changes Hydro-processing	ND	ND	ND	ND	Huang et al. (2019)
25	ND)	ND	Co-combustion with textile dyeing sludge (TDS) under varied blend ratios and heating rates	ND	Dynamic thermal degradation between 200 °C and 1000 °C, with peak interactions occurring between 350 °C and 600 °C	ND	ND	Slezak et al. (2019)
26	ND	ND	Steam Gasification at 800 °C–900 °C with steam concentrations from 10 to 50 vol%	ND	Temperature: 800 °C–900 °C, Steam concentration: 10–50 vol%	ND	Syngas (H_2_ + CO)	Berglund et al. (2024)
27	Oil Shale Semi-Coke (OSS)	ND	Co-pyrolysis with varying SMS/OSS mass ratios (0:1, 1:4, 1:1, 4:1, 1:0) at 470 °C	ND	Lab-scale pyrolysis reactor, final temperature: 470 °C, nitrogen flow rate: 200 mL/min	ND	Bio-oil	Jiang et al. (2017)

**FIGURE 5:**
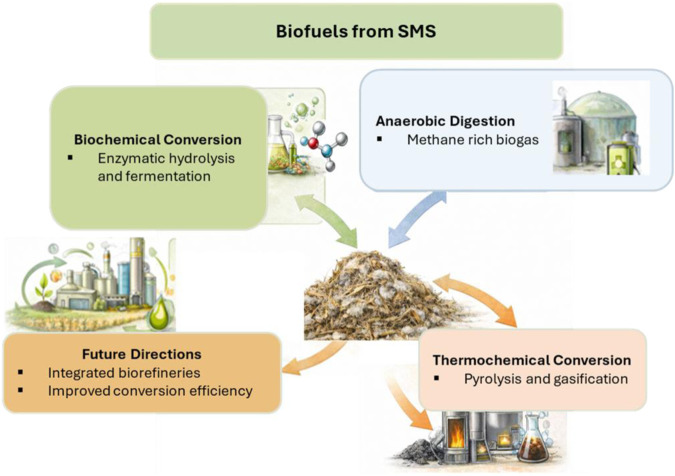
Overview of major biofuel production pathways from spent mushroom substrate (SMS). SMS can be converted through biochemical routes such as enzymatic hydrolysis followed by fermentation to produce bioethanol, or through anaerobic digestion to generate methane-rich biogas. Alternatively, thermochemical conversion processes including pyrolysis and gasification produce bio-oil, syngas, and other energy carriers.

### 4.1 Biochemical conversion – ethanol and biobutanol production

SMS substrates for biochemical conversion for ethanol and biobutanol production included hardwood chips, wheat straw, rice straw, and brewers’ spent grain. Pretreatment methods played a critical role in breaking down lignocellulosic structures to enhance enzymatic saccharification. Biological pretreatment using white-rot fungi, such as Shiitake (*L. edodes*), demonstrated significant success in saccharification yields, achieving 84% reducing sugar, which directly correlates to ethanol production efficiency (Table 4, Entry 1) (Yin Y. et al., 2025) Conversely, Reishi (*Ganoderma lucidum*) yielded a lower saccharification rate of 33.50% under similar pretreatment conditions (Table 4, Entry 2) (Yin Y. et al., 2025) When *Pleurotus ostreatus* was used under the same conditions, saccharification performance was poor (Table 4, Entry 3) (Yin Y. et al., 2025) (All three entries originate from the same comparative study) Hybrid approaches, such as microwave-, alkali-, and ultrasound-assisted pretreatments, also proved effective for SMS substrates, facilitating enhanced biofuel production. For instance, SMS derived from *P. ostreatus* processed with these hybrid techniques achieved notable outcomes in biobutanol production (Table 4, Entry 4) (Gao et al., 2021)

The enzymatic conversion of SMS utilizes enzyme blends like Cellic CTec2, comprising cellulases, β-glucosidases, and hemicellulases. Saccharification efficiency depends heavily on enzyme activity, substrate composition, and operational conditions. Under optimized settings of 50 °C, pH 5.2, and 100 CMC-ase units per gram of biomass, SMS from Shiitake demonstrated high sugar yields, supporting ethanol production as a primary biofuel outcome (Table 4, Entry 1) (Kumar et al., 2021) Biological SMS using *Clostridium thermocellum* supplemented with recombinant β-glucosidases additionally facilitated hydrogen generation, yielding 50.6% total reducing sugars after 168 h fermentation (Table 4, Entry 5) (Ryden et al., 2017)

Overall, biochemical conversion pathways demonstrate that the effectiveness of SMS as a biofuel feedstock depends strongly on the fungal species used for pretreatment, the extent of lignocellulose degradation, and the enzymatic system applied during saccharification. Among studied species, *Lentinula edodes*–treated SMS shows the most promising ethanol yields, while engineered enzymatic systems and microbial consortia such as *C. thermocellum* broaden the portfolio of attainable biofuels beyond ethanol to include biobutanol and hydrogen. This highlights that SMS performance can be tailored through careful selection of fungal strain, enzyme blend, and process optimization.

### 4.2 Anaerobic digestion – methane and biogas production

Beyond ethanol, SMS also facilitates the production of other biofuels such as methane and biogas. For methane generation, SMS combined with brewers’ spent grain or livestock manure showed enhanced yields during anaerobic digestion under mesophilic conditions (35 °C) (Table 4, Entries 6–7, 11) (Koesoemadinata et al., 2023; Moreira et al., 2020) Similarly, integrated multi-step pretreatment of rice straw and rice husk led to methane production (Table 4, Entries 8–9) (Devi et al., 2022)

Electrokinetic-assisted anaerobic digestion further demonstrated SMS’s capability in biogas production, showcasing efficiency in sugar-mill wastewater supplementation (Table 4, Entry 10) (Devi et al., 2022) Co-digestion trials combining *Flammulina velutipes*, *P. eryngii* var. *tuoliensis*, and *P. eryngii* with dairy manure under varying substrate ratios (1:1, 3:1, 1:3) achieved stable methane generation in 49-day mesophilic batch tests (Table 4, Entry 12) (Xu et al., 2024c) Likewise, *Pleurotus florida* SMS derived from waste paper produced methane following high-pressure briquetting and 60-day anaerobic digestion at 37 °C (Table 4, Entry 13) (Ravlikovsky et al., 2025)

Anaerobic digestion studies highlight the adaptability of SMS as a co-substrate for methane and biogas generation. The addition of nutrient-rich materials such as brewers’ spent grain, livestock manure, or sugar-mill effluent enhances microbial activity and improves gas yields under mesophilic conditions. Pretreatment strategies such as integrated alkaline-thermal steps promote partial delignification, improving digestibility and methane productivity. These results confirm that SMS can serve both as a primary and supplemental feedstock in biogas systems.

### 4.3 Thermochemical conversion – bio-oil, syngas, bio-coke, solid fuels, and activated carbon

Thermochemical pyrolysis under high-temperature CO_2_ environments generated syngas rich in hydrogen and carbon monoxide, showcasing SMS’s adaptability for advanced energy systems (Table 4, Entry 14) (Luo et al., 2018) Pyrolysis processes of SMS mixed with oil shale semi-coke produced bio-oil, emphasizing SMS’s potential for advanced biofuel applications (Table 4, Entry 27) (Suresh et al., 2024)

SMS has also been successfully densified into bio-coke and solid fuel pellets, providing renewable alternatives for combustion energy. High-pressure densification processes (21.7 MPa) ensured mechanical durability and enhanced combustion performance (Table 4, Entries 15–17) (Aniza et al., 2022; Lee T. et al., 2024; Sudhakar et al., 2021) Thermomechanical pelletization under pressures of 200 MPa generated solid biofuel pellets from SMS substrates such as paddy straw and compost, offering low-emission bioenergy solutions (Table 4, Entries 19–20) (Alves et al., 2021)

Microwave-assisted torrefaction and pyrolysis with catalytic additives such as MgO and ferric ammonium sulfate further enhanced bio-oil production efficiency (Table 4, Entry 18) (Wang et al., 2025) Steam co-gasification of Lentinula edodes–derived SMS blended with Erianthus or Japanese cedar produced high-yield syngas under 800 °C–900 °C with an optimized steam-to-carbon ratio (Table 4, Entry 21) (Lin et al., 2015) Similarly, direct steam gasification at 800 °C–900 °C with variable steam concentrations (10–50 vol%) generated hydrogen-rich syngas (Table 4, Entry 26) (Mo et al., 2024)

Activated carbon production was achieved through dual processing involving carbonization and flue gas activation of Agaricus bisporus SMS (Table 4, Entry 22) (Wang J. et al., 2024) Other advanced hybrid methods, such as thermochemical and microwave-assisted hydro-processing (Table 4, Entry 24) (Slezak et al., 2019), co-combustion with textile dyeing sludge at 200 °C–1000 °C (Table 4, Entry 25) (Berglund et al., 2024), and briquetting of sugarcane bagasse using Pleurotus ostreatus var. Florida (Table 4, Entry 23) (Huang et al., 2019) further demonstrate the broad utility of SMS in clean energy generation and solid fuel formulation.

Thermochemical transformation pathways expand the valorization potential of SMS beyond biochemical routes, enabling the production of diverse energy carriers such as syngas, bio-oil, and densified solid fuels. The process efficiency depends on factors including temperature, catalyst type, and gas composition, with CO_2_ or steam atmospheres favoring hydrogen-rich syngas yields. Densification and pelletization technologies convert heterogeneous residues into high-density fuels with superior mechanical durability and combustion stability, while co-pyrolysis and catalytic torrefaction optimize oil yield and quality. The conversion of SMS to activated carbon and fuel briquettes underscores its multifunctional value as both an energy and materials precursor. These findings affirm that thermochemical strategies provide an efficient, flexible, and scalable route for converting SMS waste into high-value energy products, advancing both waste minimization and circular bioeconomy objectives.

### 4.4 Future directions for SMS-based biofuel production

The future of spent mushroom substrate (SMS) in biofuel production is evolving rapidly, with several key directions emerging from recent research. The global mushroom industry’s continued expansion has created an abundant and underutilized feedstock supply for renewable energy generation. Considering that approximately 5 kg of SMS is produced for every 1 kg of mushrooms cultivated, this growing biomass stream presents both a challenge in waste management and an opportunity for sustainable energy conversion (Mo et al., 2024).

Technological innovations in bioconversion processes show remarkable promise, particularly in the area of bioethanol production. Recent studies have achieved ethanol yields as high as 186.9 g/kg dry matter using furfural-resistant yeasts, demonstrating the potential of advanced microbial systems to overcome inhibitory effects commonly associated with lignocellulosic hydrolysates (Xu et al., 2024c). These developments suggest a future where SMS-based biofuel production becomes increasingly cost-effective, energy-efficient, and environmentally sustainable. Moreover, mushroom cultivation itself serves as a form of biological pretreatment, partially degrading lignin and enhancing enzymatic accessibility for subsequent hydrolysis, which significantly improves downstream conversion efficiency and overall process economics.

The field is steadily moving toward more sophisticated approaches to waste valorization, where SMS is no longer viewed as a disposal problem but as a valuable component of the circular bioeconomy. Integration of mushroom cultivation with biorefinery concepts enables a continuous cycle in which agricultural residues feed mushroom growth, and the resulting SMS is transformed into renewable fuels and value-added products. This synergy has the potential to address both global food and energy demands while reducing environmental burdens.

Taken together, research on SMS-derived biofuels demonstrates that this abundant agro-industrial residue can be effectively transformed into multiple forms of renewable energy through complementary biochemical, thermochemical, and anaerobic processes. Biological and enzymatic pretreatments enhance sugar liberation for ethanol and hydrogen production, while co-digestion strategies optimize methane yields and waste stability. Thermochemical conversion further extends SMS utilization to high-density solid fuels and hydrogen-rich syngas. By integrating these conversion pathways within a circular biorefinery framework, SMS valorization provides a sustainable route for reducing waste, producing clean energy, and advancing low-carbon agricultural systems. Ultimately, the future success of SMS-based biofuel production will depend on continued technological innovation, improved pretreatment strategies, enzyme and microbial engineering, and the establishment of standardized protocols for assessing fuel yield and quality. By aligning bioenergy production with sustainable waste management practices, SMS valorization can serve as a model for circular, low-carbon bioprocessing systems that transform agricultural residues into clean energy and functional materials.

## 5 Valorization of spent mushroom substrate: materials from SMS

**FIGURE 6:**
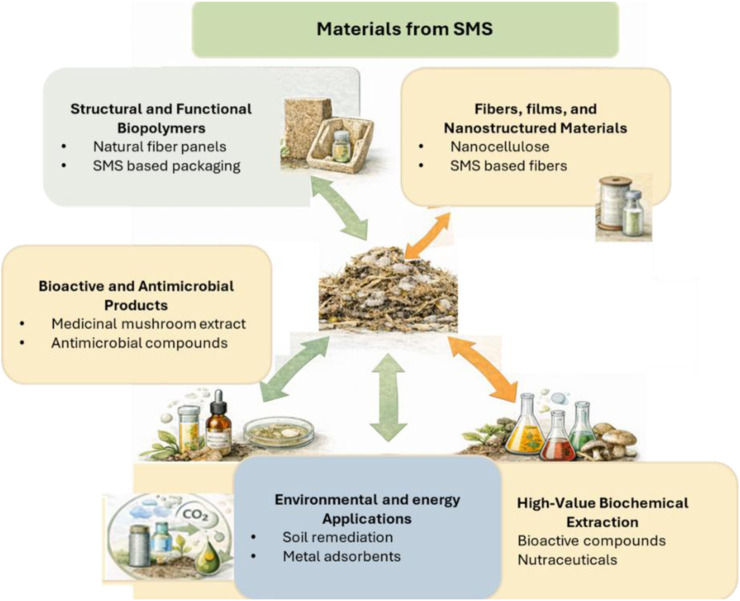
Overview of material-based valorization pathways for spent mushroom substrate (SMS). SMS can be converted into structural biopolymers and packaging materials, fibers and nanostructured materials such as nanocellulose, bioactive and antimicrobial products, and high-value biochemical extracts.

Once regarded merely as agricultural waste, SMS now underpins a growing portfolio of applications that transform this by-product into high-value materials for construction, nanotechnology, biomedicine, catalysis, and environmental remediation. Its inherent composition, cellulose, hemicellulose, lignin, proteins, and chitin, enables multiple valorization pathways aligned with circular bioeconomy principles. The reviewed studies (Table 5, Entries 1–19 and schematic Figure 6) demonstrate the diverse potential of spent mushroom substrate as a renewable and multifunctional raw material.

**TABLE 5:** Materials from SMS.

#	Application	Key Findings/Features	Ref
1	Formaldehyde-free mycelium composites	SMS served as a natural binder for particleboard substitution; high strength, antimicrobial, non-toxic adhesive alternative	Ravlikovsky et al. (2025)
2	Fibrillated biomaterials	SMS converted into non-toxic fibrillated fibers for sustainable packaging; lower energy demand than wood pulp	Ryden et al. (2017)
3	AgCl nanoparticles	SMS extract acted as a green reducing agent; nanosilver displayed strong antibacterial activity	Devi et al. (2024)
4	Feed additive (*Cordyceps militaris* SMS)	Enhanced fish immunity; served as antibiotic-free aquaculture feed supplement	Brahma et al. (2024)
5	Nanocatalyst for biodiesel	*Ganoderma lucidum* SMS transformed into CSA/BaO@K_2_CO_3_ catalyst; >94% yield, reusable	Li et al. (2024)
6	Magnetic photocatalyst (Fe_3_O_4_/MoS_2_–O@BC)	SMS-derived biochar composite degraded 90% levofloxacin and showed >99% antibacterial efficiency	Halim and Razali (2023)
7	Binder for peat-moss pellets	SMS improved pellet hardness and thermal performance; slightly reduced water resistance	Barden Schallemberger et al. (2021)
8	Textile dye decolorization	SMS immobilized laccase; enhanced azo-dye adsorption and enzymatic degradation	Iuama et al. (2022)
9	Eco-bricks	Incorporation of SMS yielded low-impact construction blocks with reduced CO_2_ footprint	Dhanabalan and Ayyasamy (2021)
10	Biosorbent for Fe^2+^ removal	*Pleurotus florida* SMS removed Fe^2+^ from groundwater (≈100% efficiency at neutral pH)	Chen et al. (2021)
11	Bio-based polyurethane	Liquefied SMS used to coat urea; extended N-release from 5 → 28 days	Katas et al. (2021)
12	Ag nanoparticles in hydrogels	SMS served as reducing agent; AgNP-gelatin hydrogel formed antimicrobial wound dressings	Khoo et al. (2020)
13	Natural bio-boards	*Ganoderma lucidum* SMS replaced synthetic adhesives; high bonding strength and fire resistance	Ariff et al. (2017)
14	Cellulase fermentation feedstock	SMS supported enzyme production without chemical pretreatment; 1.5× higher CMCase activity	Beckers et al. (2019)
15	Biorefinery colloids	SMS fractionated into carbohydrate surfactants and soluble lignin nanocarriers	Wang et al. (2018)
16	Fungal sterol recovery	Ultrasound-assisted extraction from shiitake SMS yielded 151.6 mg ergosterol eq./100 g DW.	Siracusa et al. (2017)
17	PCB-contaminated soil remediation	SMS used as bulking agent in biopiles; 94% PCB depletion in 8 months	Xu et al. (2017)
18	Probiotic nutrient solution	Fermented *Flammulina velutipes* SMS produced safe nutrient-rich probiotic liquid	Dang et al. (2020)
19	SMS as aggregate in mortar	Up to 12.5% SMS replacement reduced CO_2_ emissions and cost in lightweight masonry	Domínguez-Gutiérrez et al. (2022)

Depending on the processing approach (mechanical, biological, or thermal), SMS can be converted into biocomposites (Entries 1, 9, 13, 19), fibers and nanostructured materials (Entries 2, 5, 6, 15), bioactive or antimicrobial products (Entries 3, 4, 11, 12, 18), environmental and energy materials (Entries 7, 8, 10, 17), and biochemical feedstocks (Entries 14, 16). These applications highlight SMS as an abundant, low-cost, and sustainable resource capable of replacing petrochemical-based or toxic raw materials in a range of industrial and environmental technologies.

### 5.1 Structural and functional biocomposites

SMS has been widely used as a base material for biocomposite production. Formaldehyde-free mycelium composites were produced from SMS mixed with chitin-rich crayfish shells through hot pressing, generating materials with improved strength (up to 1383 MPa), hydrophobicity, and antimicrobial activity while reducing greenhouse gas emissions by over 80% (Table 5, Entry 1). Similarly, bio-boards made from *Ganoderma lucidum* SMS were fabricated using the intrinsic mycelium as a binder, eliminating synthetic adhesives. These bio-boards achieved internal bonding strengths up to 2.51 MPa, exceeding U.S. and Chinese industrial standards, and exhibited strong water and fire resistance (Table 5, Entry 13). SMS has also been applied in the construction sector. *Lentinula edodes* SMS was incorporated into eco-bricks that maintained a compressive strength of 0.8 MPa and contained no detectable toxic elements. Although the water resistance was lower than conventional bricks, the material was suitable for non-structural uses, reducing raw material and energy consumption (Table 5, Entry 9). Furthermore, SMS has been used as a partial replacement for fine aggregates in concrete mortar, achieving cost reduction and up to 12.5% SMS incorporation with balanced mechanical and durability properties (Table 5, Entry 19). These examples demonstrate that SMS-derived biocomposites can rival or exceed conventional particleboard or mortar in strength while drastically lowering embodied carbon.

### 5.2 Fibers, films, and nanostructured materials

SMS can be processed into fibrillated biomaterials and nanostructured composites. When used as a biologically pretreated lignocellulosic resource, SMS was fibrillated at high solids content with low energy demand (1.7 kWh kg^-1^), forming a strong, antioxidant-active network suitable for sustainable packaging films (Table 5, Entry 2). A biorefinery approach separated SMS into carbohydrate- and lignin-rich fractions. The carbohydrate fraction exhibited surfactant properties, while the lignin fraction was used to fabricate biodegradable nanocarriers via inverse miniemulsion polymerization (Table 5, Entry 15). SMS has also been utilized in catalysis and photocatalysis. *Ganoderma lucidum* SMS was transformed into a nanocatalyst (CSA/BaO@K_2_CO_3_) via wet impregnation, achieving a biodiesel yield of 94.36% under optimized conditions (Table 5, Entry 5). In another study, SMS-derived biochar was combined with Fe_3_O_4_/MoS_2_–O to create a magnetic photocatalyst (Fe_3_O_4_/MoS_2_–O@BC) capable of degrading 90.64% of levofloxacin under visible light and exhibiting >99% antibacterial efficiency against *Staphylococcus aureus* and *Escherichia coli* (Table 5, Entry 6). Such conversion of biologically conditioned SMS into nano-structured catalysts and fibers exemplifies a biorefinery pathway bridging packaging, catalysis, and energy materials.

### 5.3 Bioactive and antimicrobial products

SMS has been used as a biological reducing agent and as a source of antimicrobial compounds. SMS extract served as a low-cost reagent for producing silver chloride (AgCl) nanoparticles with an average size of 8.3 nm. These nanoparticles displayed strong antibacterial activity against *Ralstonia solanacearum*, attributed to cell membrane disruption and reactive oxygen species generation (Table 5, Entry 3). Similarly, silver nanoparticles synthesized using SMS were incorporated into gelatin hydrogels, yielding wound dressings with antibacterial and anti-biofilm properties against *S. aureus*, *B. subtilis*, *P. aeruginosa*, and *E. coli* (Table 5, Entry 12).

Beyond nanomaterials, SMS has shown biological activity in animal feed and agriculture. The SMS of *Cordyceps militaris* was used as an in-feed antibiotic alternative for *Labeo rohita* fish. At inclusion levels of 2%–3%, survival rates increased significantly due to the immunostimulant effects of SMS constituents, including cordycepin and adenosine (Table 5, Entry 4). In a related agricultural application, liquefied SMS was used to develop a bio-based polyurethane coating for controlled nutrient release from urea fertilizers. The modified coating reduced water absorption (from 18.27% to 3.27%) and prolonged nitrogen release from 5 to over 28 days (Table 5, Entry 11). SMS fermentation has also been explored for producing probiotic nutrient solutions rich in sugars, proteins, and minerals, achieving safe microbial and heavy metal profiles (Table 5, Entry 18). These studies show SMS’s dual role as a bio-reductant and functional additive, offering antibiotic-free routes to antimicrobial and agricultural innovation.

### 5.4 Environmental and energy applications

In environmental remediation, SMS exhibits high potential as a biosorbent and enzymatic material. SMS naturally contains laccase, lignin peroxidase, and manganese peroxidase, enabling simultaneous adsorption and enzymatic degradation of textile azo dyes. The combined mechanisms achieved 57.22% adsorption at pH 8% and 14.18% enzymatic degradation at pH 4 (Table 5, Entry 8). *Pleurotus florida* SMS effectively removed Fe^2+^ from groundwater, reaching 100% removal efficiency at neutral pH and ambient temperature (Table 5, Entry 10). In soil bioremediation, SMS functioned as a bulking agent promoting microbial degradation of polychlorinated biphenyls (PCBs), achieving 94.1% removal in a pilot-scale biopile over 8 months (Table 5, Entry 17).

In energy-related uses, SMS has been incorporated into biofuel pellets as a natural binder. When mixed with peat moss, the addition of SMS increased pellet hardness (up to 45.33 Durometer) and durability (PDI >97.5%), while maintaining acceptable water resistance (Table 5, Entry 7). The multifunctionality of SMS in adsorption, enzymatic degradation, and pelletization positions it as a low-cost platform for both pollution control and decentralized bioenergy.

### 5.5 High-value biochemical extraction

SMS also serves as a source of valuable biochemicals. Ultrasound-assisted extraction from spent *shiitake* substrate yielded fungal sterols, mainly ergosterol, at 151.62 mg ergosterol equivalents per 100 g dry weight. The extracts showed moderate antitumor activity (Table 5, Entry 16). In enzyme production, *Pleurotus pulmonarius* SMS was used directly as a delignified fermentation feedstock for cellulase production under solid-state conditions, achieving CMCase and β-glucosidase activities of 171.21 U/g and 6.83 U/g, respectively, without the need for chemical pretreatment (Table 5, Entry 14). These findings emphasize the untapped potential of SMS as a secondary bioprocess feedstock capable of supplying enzymes, sterols, and other biochemicals traditionally derived from costly microbial cultures.

Material valorization pathways highlight SMS as a multifunctional, renewable resource that integrates material production, bioactivity, and environmental remediation within a single feedstock platform. Whether transformed into structural composites, catalytic nanomaterials, or biochemical precursors, SMS demonstrates versatility across industrial sectors while diverting significant organic waste from disposal. Continued research aimed at optimizing process scalability, property tailoring, and end-of-life recyclability will accelerate SMS adoption in green manufacturing and circular bioeconomy initiatives.

## 6 Efficient valorization of SMS through composting and vermitechnology

### 6.1 Overview and significance

Among the various strategies for SMS valorization, aerobic composting and vermitechnology (vermicomposting or vermiculture-based biotransformation) have emerged as two of the most practical biological pathways for converting this lignocellulosic residue into value-added agricultural products. In these processes, microbial composting and earthworm-mediated vermicomposting transform unstable spent mushroom substrate into a mature, stable, and nutrient-rich soil amendment suitable for agricultural use. Both approaches convert a high-volume cultivation by-product into stabilized organic amendments that can be applied as soil improvers or fertilizers while simultaneously reducing disposal burdens and environmental risks associated with uncontrolled accumulation or landfilling (Ma et al., 2023). Over the past decade, studies ranging from laboratory-scale experiments to pilot and industrial systems have demonstrated that SMS can be effectively integrated into existing organic waste management infrastructures (Ma et al., 2023).

In addition to stabilizing the material, composting and vermitechnology substantially reduce the physical mass and volume of SMS during processing. Pilot-scale trials treating approximately 800 kg of fresh SMS per batch reported reductions exceeding 53% in fresh mass and about 58% in total volume during stabilization. In parallel with these physical reductions, processed SMS typically exhibits improved agronomic properties, including increased nutrient availability, higher cation exchange capacity (CEC), and reduced phytotoxicity (Ma et al., 2023).

Process performance can be further influenced by microbial additives or inoculants that accelerate lignocellulosic degradation. For instance, *Bacillus*-based inocula combined with controlled turning have been reported to support thermophilic composting cycles of approximately 30 days in small-scale reactors while maintaining high germination index values (Yin J. et al., 2025). Similarly, lignocellulose-degrading microbial consortia such as PLC-8 have been shown to accelerate heat development and alter greenhouse gas emission patterns in larger composting piles, highlighting the interaction between inoculation strategies and operational parameters such as the carbon-to-nitrogen ratio (European Union, 2025)

While numerous studies demonstrate the technical feasibility of SMS composting, large-scale implementation depends not only on process performance but also on regulatory compliance and product marketability. In many regions, the commercialization of compost products requires adherence to standards governing contaminants, pathogens, and product safety. For example, regulatory frameworks in the European Union establish quality requirements for materials marketed as organic soil improvers or growing media, including microbiological safety criteria such as the absence of *Salmonella* in 25 g samples and limits for indicator organisms such as *E. coli* or Enterococcaceae, as well as maximum allowable concentrations of heavy metals including Cd and Pb (Iglesias et al., 2025). Additionally, life-cycle assessment and techno-economic analyses highlight that environmental performance remains sensitive to transportation logistics, processing conditions, and the composition of the original cultivation substrate (Code of federal regulations, 2025).

These findings indicate that efficient SMS valorization through composting and vermitechnology should be viewed as a systems-level design challenge rather than a single operational choice. Because stabilization efficiency is closely linked to substrate physicochemical composition and the biological processes governing organic matter degradation, understanding SMS composition and its influence on microbial and earthworm-driven transformations is essential for optimizing these pathways.

### 6.2 SMS composition and mechanism of composting and vermitechnology

The chemical composition of SMS strongly influences its suitability for composting and vermitechnology. Spent mushroom substrate typically contains substantial lignocellulosic fractions derived from the original cultivation materials, including cellulose, hemicellulose, and lignin, along with high levels of organic matter. Cellulose and hemicellulose provide the primary substrates for microbial hydrolysis and fermentation, while lignin forms a more recalcitrant aromatic matrix that limits enzymatic accessibility and slows overall decomposition rates. Nutrient concentrations, particularly nitrogen, phosphorus, and potassium, vary widely depending on substrate formulation and cultivation practices (Ravlikovsky et al., 2024). Physicochemical properties such as pH and electrical conductivity also span broad ranges, reflecting differences in feedstock composition and mineral amendments used during mushroom production. These parameters influence microbial community structure and enzymatic activity, thereby affecting the rate and pathway of organic matter mineralization. In some cultivation systems, especially those associated with *Agaricus* substrates, elevated salinity may occur and can limit direct agricultural application for salt-sensitive crops (Ravlikovsky et al., 2024).

Moisture content is another critical parameter affecting aeration, structural stability, and microbial activity during biological stabilization. Adequate moisture facilitates microbial metabolism and enzyme diffusion within the compost matrix, whereas excessive moisture can reduce pore space, restrict oxygen transfer, and promote anaerobic microenvironments that inhibit efficient aerobic decomposition. Because of the substantial variability in these properties, efficient SMS valorization generally requires preliminary characterization of incoming material, including moisture content, salinity, and carbon-to-nitrogen (C:N) ratio, followed by adjustment of feedstock mixtures through bulking agents or nutrient amendments to achieve optimal composting or vermicomposting conditions (Ravlikovsky et al., 2024).

Aerobic composting stabilizes organic residues through microbial oxidation and enzymatic depolymerization of organic matter. During this process, diverse microbial communities produce extracellular enzymes such as cellulases, hemicellulases, and ligninolytic oxidases that progressively break down polysaccharides and aromatic polymers into simpler compounds that can be mineralized or incorporated into humic substances. In SMS, readily degradable components have already been partially consumed during mushroom cultivation, leaving a relatively recalcitrant lignocellulosic matrix. Consequently, effective composting requires adequate oxygen transfer, microbial accessibility, and balanced nutrient availability to sustain decomposition of residual carbohydrates and structural polymers. Microbial respiration generates metabolic heat, leading to the thermophilic phase that enhances enzymatic activity, accelerates organic matter turnover, and suppresses pathogenic microorganisms. The thermophilic phase that typically develops during composting accelerates degradation and contributes to pathogen reduction (Yin J. et al., 2025).

Vermitechnology adds an additional biological dimension to SMS stabilization through the activity of earthworms and their associated microbial communities (Ma et al., 2023).Earthworms fragment organic particles and stimulate microbial processing through gut-associated transformations, promoting nitrification, improved nutrient availability, and enhanced humification compared with conventional composting alone. Mechanical fragmentation by earthworms increases the surface area available for microbial colonization, while enzymatic and microbial processes in the earthworm gut transform organic residues into more bioavailable compounds that are subsequently released in nutrient-rich casts. Because earthworms cannot tolerate thermophilic temperatures, vermicomposting typically operates under mesophilic conditions (Ma et al., 2023). As a result, many SMS processing strategies employ a two-stage system in which thermophilic composting provides initial stabilization and sanitation, followed by vermicomposting to further enhance nutrient transformations and product quality (Ravlikovsky et al., 2024). Although substrate composition determines the biochemical potential of SMS for microbial degradation, stabilization efficiency ultimately depends on operational conditions within the composting system. Key parameters, including temperature, moisture content, aeration, and carbon-to-nitrogen (C:N) ratio, must therefore be carefully managed to support efficient decomposition. These factors regulate microbial metabolic activity, enzyme production, oxygen availability, and nutrient balance, collectively determining the rate of organic matter mineralization and the formation of stable humified products.

### 6.3 Operational parameters affecting SMS composting

Efficient stabilization of SMS requires careful control of several operational parameters. Temperature is a key driver of microbial activity and sanitation during composting. Effective degradation typically occurs during the thermophilic phase when temperatures exceed ∼55 °C (Yin J. et al., 2025). Regulatory frameworks often specify time–temperature regimes to ensure pathogen reduction (Lin et al., 2022). For example, windrow composting guidelines commonly require maintaining temperatures above ∼55 °C for extended periods with periodic turning. In contrast, some pilot-scale SMS vermireactor systems operate under ambient conditions without active temperature control, emphasizing moisture management and processing time rather than thermophilic heating (Ma et al., 2023).

The carbon-to-nitrogen (C:N) ratio is another critical factor governing microbial metabolism. Ratios in the range of ∼25:1–30:1 are commonly targeted in SMS composting systems to balance microbial growth with nitrogen conservation (European Union, 2025). Fresh SMS may exhibit highly variable C:N ratios depending on the cultivation substrate, making recipe adjustments through manure or other nitrogen-rich amendments common practice (Ravlikovsky et al., 2024). Moisture content is typically maintained around ∼50–60% in controlled systems, although higher values may be tolerated when sufficient structural aeration prevents anaerobic conditions (Lin et al., 2022; Ma et al., 2023; Yin J. et al., 2025).

Aeration and turning strategies further influence compost performance. Regular turning enhances oxygen diffusion, redistributes heat, and prevents localized anaerobic zones. Turning schedules vary across systems, ranging from frequent agitation during thermophilic phases to weekly turning in longer-duration processes (Ma et al., 2023; Yin J. et al., 2025; European Union, 2025). Bulking agents such as straw are often incorporated to improve porosity and structural stability, particularly when SMS exhibits high moisture content or fine particle structure (Ansari et al., 2022; Yin J. et al., 2025). In addition, microbial inoculants, including *Bacillus*-based formulations and lignocellulose-degrading consortia, have been explored to accelerate decomposition and influence greenhouse gas emission patterns (Yin J. et al., 2025; European Union, 2025).

Reported composting durations vary depending on reactor configuration, management intensity, and feedstock composition, typically ranging from roughly one to 4 months in composting systems (Yin J. et al., 2025). Vermicomposting processes generally require slightly longer stabilization periods, often on the order of several weeks to a few months (Meng et al., 2018).

### 6.4 Product quality, maturity indicator, and environmental considerations

Assessment of compost maturity typically relies on a combination of physicochemical and biological indicators. Organic matter mineralization is reflected by decreasing organic carbon and increasing ash content, while nitrogen transformations, particularly declining ammonium and increasing nitrate concentrations, provide additional evidence of microbial stabilization (Ma et al., 2023).

Biological assays such as germination index tests and plant growth trials are widely used to evaluate phytotoxicity and compost maturity. However, germination index alone may not capture all maturity-related effects, particularly those associated with insoluble organic fractions. For example, studies on co-composting systems involving SMS and other organic residues have shown that plant growth trials can serve as integrative indicators linking seedling performance to parameters such as total organic carbon, C/N ratio, nitrogen forms, available potassium, and lignocellulosic components (Environment Agency, 2025) Some SMS composting systems report germination indices exceeding 100%, indicating enhanced plant growth potential after stabilization (Yin J. et al., 2025). Increased cation exchange capacity (CEC) is also commonly observed during maturation and reflects progressive humification (Ma et al., 2023).

Despite these improvements, several constraints may affect the agricultural use of SMS-derived compost. Elevated salinity is among the most frequently reported limitations; although composting and vermicomposting can reduce electrical conductivity, levels may remain high for salt-sensitive crops. Co-composting with manure or other organic wastes may also introduce trace metals that must remain within regulatory limits for soil amendments (Iglesias et al., 2025; Environment Agency, 2025; Nordahl et al., 2023).

Composting systems also produce gaseous emissions, including carbon dioxide, methane, nitrous oxide, ammonia, and volatile organic compounds (US Composting Council, 2021). Emission levels are strongly influenced by aeration, moisture conditions, and nitrogen transformations, highlighting the importance of appropriate process control to minimize environmental impacts.

### 6.5 Operational considerations and scale-up

Practical implementation of SMS composting requires addressing several operational challenges. High moisture content and fine particle structure can promote compaction and oxygen limitation, creating anaerobic zones that increase odor generation and methane emissions. These effects are commonly mitigated through moisture adjustment and the use of structural bulking agents that improve pile permeability and aeration (Ansari et al., 2022).

Salinity management is another important consideration, particularly for horticultural applications with salt-sensitive crops. Even after stabilization, electrical conductivity may remain elevated due to salts present in the original cultivation substrate. In such cases, dilution with low-salinity materials, extended curing, or application in field systems with greater salt tolerance may be required (Ma et al., 2023).

Industrial-scale systems demonstrate that SMS can be processed efficiently at large volumes using technologies such as agitated beds, aerated static piles, or in-vessel reactors capable of handling hundreds of tons of organic material. These systems improve process control and throughput but require greater infrastructure investment, operational oversight, and regulatory permitting (Ansari et al., 2022; Yin J. et al., 2025).

Life-cycle assessments indicate that converting SMS into soil amendments can provide environmental benefits by recycling nutrients and organic matter within agricultural systems and reducing reliance on synthetic fertilizers (Code of federal regulations, 2025). However, overall performance depends on factors such as transportation logistics, aeration energy use, and management of emissions or leachate.

Economic feasibility depends on operational costs associated with feedstock handling, bulking agents or amendments, labor, water management, infrastructure, and post-processing activities such as screening and product testing. Larger facilities may reduce per-ton processing costs but generally require higher capital investment and more complex permitting (Ansari et al., 2022; Ma et al., 2023). Potential revenue streams include avoided disposal fees, sales of compost or vermicompost products, and in some cases vermiculture co-products. Access to higher-value markets often requires routine product testing and certification, making quality assurance both a cost and an important factor for market access (Yu et al., 2022).

### 6.6 Comparative overview of recent studies

A comparative summary of recent research on SMS composting and vermitechnology is presented in Table 6, highlighting representative studies published within the last decade. The table emphasizes feedstock composition, processing methods, key operational parameters, and major findings relevant to SMS stabilization and nutrient recovery. Particular attention is given to pilot- and industrial-scale investigations, as well as studies examining inoculant strategies, co-composting with animal manures, and combined composting–vermicomposting systems. This synthesis provides a practical reference for identifying operational windows and knowledge gaps in the current literature.

**TABLE 6:** Summary of recent research on SMS composting and vermitechnology

Scale	Feedstock details	Method(s)	Key process parameters reported	Key findings	Ref
Pilot scale	Not provided	Composting pile	Weekly turning, 120 days	Reduction in volume, mass and nutrients; Reduction in sodium and calcium salts	Ma et al. (2023)
Pilot scale	Not provided	Vermireactor	120 days; moisture ∼85–95%; ambient/no temp control	Reduction in volume, mass and nutrients; Reduction in sodium and calcium salts; Higher concentration of nutrients than in compost; Demonstrates pilot feasibility	Ma et al. (2023)
Reactor scale	Chicken manure and *Auricularia cornea* cv. Yu Muer residue + straw	Composting box	Aerobic composting with *Bacillus* sp. (*Bacillus subtilis*, *Bacillus licheniformis*, and *Bacillus laterosporus*) added at 1%, moisture 60%; 30 days	Straw addition during cultivation improves subsequent composting of spent mushroom substrate	Yin et al. (2025b)
Pilot (∼5 m^3^)	Spent mushroom substrate (*Auricularia heimuer*) mixed with pig manure (3:1); C/N adjusted with urea; microbial inoculum PLC-8 added in some treatments	Aerobic composting	Piles manually turned when temperature exceeded ∼50 °C and periodically during cooling; composting duration ∼60 days	Microbial inoculum accelerated thermophilic phase onset, improved cellulose and hemicellulose degradation, altered greenhouse-gas emission patterns, and shifted microbial community composition toward lignocellulose-degrading taxa (e.g., *Proteobacteria* and *Ascomycota*)	(European Union, 2025)
Industrial scale	SMS from *Flammulina velutipes* operations + swine and poultry manure at 6:4 v/v; SMS-SW = spent mushroom substrate + soy waste at 6:4 v/v	Co-composting in a rectangular agitated bed composting system with controlled aeration and periodic turning	Initial moisture adjusted to ∼50–55% by water spraying; turning frequency every 2–3 days; composting duration 30 days in summer with daily turning and 60 days in winter with turning every 2 days	Manure addition changed the compost microbiota, especially the bacterial community. In SMS-SW, dominant bacteria were *Weissella paramesenteroides* and *Lactobacillus helveticus*; in SMS-PM, dominant bacteria were Thermotogaceae *sp*. and *Ureibacillus sp*. Provides real scale-up evidence (equipment + operational framework) for SMS co-composting	Ansari et al. (2022)
Pilot scale	SMS + biogas residues (BR), and pig manure (PM) mixed at 1:1:1; SMS from *Auricularia auricula* production, BR from corn-stalk-fed biogas plant, PM from swine herd	Aerobic co-composting in a concrete composting bunker with manual turning	Composting duration: 118 days; manual turning when temperature exceeded 50 °C; turning once/day during thermophilic stage and once every 5 days after temperature fell below 35 °C	Compost supported tomato seedling growth, with 80% compost substrate giving the best seedling quality; identified maturity indicators correlating with seedling quality (TOC, C/N, NH_4_^+^-N, NO_3_^−^-N, available K, lignocellulose)	Environment Agency (2025)
Laboratory scale	Mushroom waste (MW) and raw mushroom waste (RMW) derived from wheat straw (*Agaricus bisporus*) and soybean straw (*Pleurotus* sp.); treatments with effective microorganisms (EM) and vermicomposting (*Eisenia fetida*)	Aerobic composting and vermicomposting in glass-jar reactors with periodic aeration	Effective microorganisms (EM) added; moisture maintained ∼50–60%; aeration every 3 days; earthworms added after 15 days of pre-decomposition; total processing time ∼6 weeks; ambient temperature ∼25 °C–30 °C	Vermicomposting combined with EM accelerated decomposition, reduced C:N ratio, increased NPK availability, and produced mature compost faster than EM treatment alone	Meng et al. (2018)
Laboratory scale	Cow dung (CD), spent mushroom substrate (SMS), and a mixture of CD + SMS (1:2 w/w)	Aerobic composting followed by vermicomposting (two-stage process)	Pre-composting for 75 days with weekly mixing; C:N adjusted to ∼25; moisture 70%–80% during composting; substrates sieved (<5 mm); vermicomposting with *Eisenia fetida* (100 worms per 10 kg substrate) for 60 days; temperature 24 °C–32 °C; moisture 60%–70%	Co-composting SMS with cow dung and subsequent vermicomposting improved compost maturity and quality, increased nitrate and EC, reduced total carbon and C:N ratio, and enhanced microbial diversity and activity	Ruangjanda et al. (2022)
Laboratory scale	Bedding mixture containing soil, cow dung, rice husk ash, vegetable waste and spent mushroom substrate (SMS); supplemented with Azolla biomass, eggshells, fruit peel waste, or cassava pulp	Vermicomposting in plastic bucket reactors after 2-week pre-composting	Pre-composting 14 days; earthworm Eudrilus eugeniae added at ∼20 worms kg^-1^; experiment duration 45 days; drainage holes for aeratio	Agro-residue additions (especially fruit peel and cassava pulp) improved earthworm growth, enzyme activity, nutrient content, and reduced C/N ratio of the vermicompost	Kong et al. (2022)
Laboratory scale	Pig manure and corn stalks (85:15 wet weight) with additives: biochar, CaMgP fertilizer, or spent mushroom substrate (SMS)	Aerobic composting in a 60 L forced-aeration laboratory reactor	Composting duration 49 days; continuous aeration with controlled ventilation rate; temperature monitored by sensors; additive treatments (Biochar, Calcium magnesium phosphate fertilizer (CaMgP))	Additives accelerated organic matter degradation, promoted humification, reduced volatile fatty acids, and enhanced heavy-metal passivation; SMS most effective for Cd, Cr, and Pb immobilization	Liu et al. (2020)
Laboratory scale	Pig manure and corn stalks (85:15 wet weight) with additives: biochar, CaMgP fertilizer, or spent mushroom substrate (SMS)	Aerobic composting in a 60 L forced-aeration laboratory reactor	Composting duration 49 days; aeration 0.3 m^3^ h^-1^ (days 1–15) then 0.2 m^3^ h^-1^ (days 16–49); turning and sampling	Additives accelerated compost maturity and reduced gaseous emissions. CaMgP most effectively reduced NH_3_ and H_2_S emissions, biochar improved compost maturity (highest GI), and SMS reduced N_2_O emissions (∼37%) and sulfur odor compounds	Zheng et al. (2014)
Industrial system modeling (LCA/LCC)	SMS derived from *Agaricus bisporus* cultivation; fresh substrate composed of mixtures of wheat straw, chicken manure, horse manure, gypsum, lime, and peat casing	Life Cycle Assessment (LCA) and Life Cycle Costing (LCC) modeling of SMS production and recomposting for use as soil improver	Functional unit: 1 ton SMS; system boundary cradle-to-gate-to-grave; includes fresh substrate composting, pasteurization, mushroom cultivation, re-composting, and agricultural land application; economic allocation between mushrooms and SMS	SMS used as soil improver showed substantially lower environmental impacts than mineral fertilizer baseline, especially for land use and fossil resource depletion; recomposting stage dominates environmental impacts	Code of federal regulations (2025)

### 6.7 Recommended operating protocols and vermitechnology pathways

Based on recent studies, practical operating windows can be proposed for SMS composting. For small-scale or on-farm systems, the main goal is to stabilize SMS for safe agricultural use or blending into potting substrates. Initial characterization should include moisture, electrical conductivity, and an estimated C:N ratio, since SMS composition varies widely and fixed recipes may not perform well. Structure can be improved by adding coarse bulking agents such as straw or other lignocellulosic residues. Where nitrogen is limiting, manure or other N-rich materials may enhance microbial activity, although ammonia losses should be managed.

Typical operating targets are initial moisture of about 50%–60% and a C:N ratio near 25–30:1. Piles should be large enough to retain heat while maintaining aeration. Turning is usually guided by temperature, with more frequent turning during thermophilic phases and less during maturation. Where sanitation is required, systems should meet recognized time–temperature criteria, such as maintaining temperatures above 55 °C for the required duration. At the end of composting, quality assurance should include maturity testing, such as germination index assays or plant growth trials, together with electrical conductivity measurements. If salinity remains high, blending or dilution may be needed before use. While these guidelines are suitable for small-scale stabilization, industrial composting requires tighter process control and more formal quality assurance to ensure consistent performance and regulatory compliance.

Industrial composting aims to produce uniform products that meet market and regulatory standards. Large-scale systems often use agitated beds, aerated static piles, or in-vessel technologies that improve aeration and shorten processing time. Operating parameters are similar to those at smaller scale, with moisture around 50%–60% and C:N near 25–30:1. Mechanical turning or forced aeration helps maintain oxygen supply and even temperature distribution. Quality assurance programs generally include routine testing for maturity, pathogen reduction, and heavy metal content in line with regional regulations.

Sampling frequency and batch definition are also important for quality control. In addition to conventional composting, vermitechnology has emerged as a complementary option for SMS stabilization and nutrient transformation. Two main strategies are reported: vermicomposting as a finishing step after thermophilic composting, where composting provides sanitation and partial stabilization and earthworms further improve the material; and direct or hybrid vermicomposting of partially stabilized SMS. Pilot studies suggest the latter is feasible, but pathogen reduction may depend on prior pasteurization during mushroom production or on post-treatment verification.

Successful vermicomposting requires conditions favorable to earthworms. Excessive heat, ammonia, unsuitable moisture, and high salinity can reduce worm survival and activity. Careful control of substrate conditions is therefore essential when using vermitechnology for SMS valorization.

### 6.8 Research gaps and future directions

Despite significant progress in SMS valorization research, several important knowledge gaps remain. One key challenge involves quantifying trade-offs between compost maturity and greenhouse gas emissions. Studies show that microbial inoculants and C:N adjustments can simultaneously influence decomposition rates and emission profiles, but standardized evaluation frameworks are still lacking.

Salinity management also represents a critical research priority, particularly for applications in horticultural substrates. Future work should evaluate strategies such as controlled leaching, feedstock blending, and extended curing to reduce electrical conductivity while minimizing nutrient losses.

Another area requiring further investigation is pathogen and contaminant fate in vermitechnology-only systems. While regulatory frameworks define acceptable microbial limits for compost products, relatively few studies have directly evaluated whether vermicomposting alone can achieve sufficient pathogen reduction in SMS processing systems.

Finally, comprehensive life-cycle assessments comparing composting and vermicomposting pathways remain limited. Integrated environmental and economic evaluations using pilot- and commercial-scale data will be essential for determining the most sustainable strategies for SMS valorization within circular bioeconomy frameworks.

## 7 Circular economy perspectives on SMS valorization pathways

From a circular economy perspective, SMS valorization should be evaluated not only by product yield or functional performance, but also by its ability to retain material value, displace virgin resources, minimize waste generation, and enable cascading use of biomass. Because SMS is heterogeneous, wet, and geographically dispersed, the most circular route is not universal but depends on local infrastructure, end-use markets, and the feasibility of integrating multiple recovery steps within a regional system. The Table 7 below provides a comparative overview of major SMS valorization pathways, illustrating how different conversion routes contribute to circular economy objectives through waste reduction, resource substitution, and value recovery, while also identifying key trade-offs and technological constraints that influence their practical deployment.

**TABLE 7:** Circular economy analysis of spent mushroom substrate valorization routes.

Pathway	Circular value proposition	Main resource displaced	Major circularity benefit	Key limitation/trade-off
Biochar	Converts waste into stable carbon product	Activated carbon, mineral sorbents, some soil amendments	Carbon retention, pollutant removal, landfill diversion	Drying and pyrolysis energy; end-of-life of spent sorbents
Biofuels	Recovers energy from residual biomass	Fossil fuels	Waste-to-energy, reduced disposal burden	Lower value retention than materials routes
Biocomposites/boards	Preserves structural value of biomass	Synthetic boards, petrochemical binders, virgin fillers	Higher-value material substitution	Standardization, durability, moisture sensitivity
Nanomaterials/catalysts	Converts SMS into functional advanced materials	Conventional catalysts/supports	High value per unit mass	Scale-up, reproducibility, purification burden

Biochar and carbon-based materials represent a hybrid circular pathway in which SMS is diverted from disposal and converted into stable carbon products with environmental functionality. These routes can support circularity by extending carbon residence time, reducing landfill methane emissions, and substituting for mined adsorbents, activated carbons, or synthetic remediation materials. However, their circular performance depends strongly on drying demand, pyrolysis energy input, and the fate of mineral-rich residues or spent sorbents after pollutant capture.

Biofuel pathways contribute to circularity primarily through energy recovery from an unavoidable agricultural residue. Anaerobic digestion, fermentation, gasification, and densification convert residual organic matter into methane, ethanol, syngas, bio-oil, or solid fuels, thereby offsetting fossil energy use and reducing disposal burdens. Yet, these routes often occupy a lower position in biomass value hierarchies because they recover energy rather than preserve material complexity.

Materials-oriented valorization routes are especially attractive from a circular economy standpoint because they preserve more of the embedded structural and chemical value of SMS. The production of biocomposites, boards, fibers, nanostructured materials, catalysts, sorbents, and bioactive products can displace petrochemical polymers, synthetic binders, virgin fillers, and energy-intensive functional materials. These routes align with circular design principles by extending material life, enabling substitution of nonrenewable inputs, and creating higher-value applications from secondary biomass. Their main challenges include feedstock variability, standardization, durability, contamination control, and end-of-life management of the resulting products.

Composting and vermiculture-based biotransformation remain important circular pathways, particularly where local agriculture can absorb stabilized organic amendments. These approaches return nutrients and organic matter to soil, reduce unmanaged decomposition, and provide relatively low-tech solutions for decentralized SMS management. However, although composting and vermicomposting are highly relevant for nutrient cycling, they generally generate lower economic value than materials or specialty carbon products.

SMS should not be viewed through a single end-use model, but through a cascade valorization framework. Where feasible, the most circular strategy may involve sequential use: first recovering extractives, fibers, or functional fractions; then converting the remaining solids into composites, biochar, or fuels; and finally returning residual mineral or organic fractions to soil where safe and appropriate. Future progress in SMS circularity will depend on matching feedstock quality with the highest feasible value pathway, supported by TEA/LCA, regional logistics analysis, and design of integrated systems rather than isolated conversion technologies.

## 8 Conclusion

The key findings and perspectives emerging from this review can be summarized as follows:

1. SMS generation at large scale creates significant environmental and economic pressures. High moisture content, low bulk density, transportation costs, and restrictions on organic waste disposal make uncontrolled accumulation unsustainable while contributing to methane emissions, leachate formation, and localized environmental impacts.
2. SMS represents a scalable lignocellulosic platform for circular manufacturing rather than merely an agricultural residue. Its composition, containing lignocellulosic fibers, residual fungal biomass, and mineral nutrients, makes it a versatile feedstock for thermochemical conversion and materials development.
3. Biochar production is one of the most mature valorization pathways. Pyrolysis converts SMS into porous carbon materials with tunable surface chemistry and mineral composition suitable for carbon sequestration, pollutant adsorption, soil conditioning, and catalytic applications. SMS-derived biochars can serve as functional precursors for advanced carbon materials. Activation, mineral functionalization, and magnetic or photocatalytic modification enable applications in environmental remediation, antimicrobial systems, and catalytic processes.
4. SMS also demonstrates significant potential as a precursor for structural and functional materials. Examples include formaldehyde-free particleboards, mycelium-based bio-boards, fibers for sustainable packaging, eco-bricks, lightweight construction aggregates, and bio-based polymer systems. At smaller length scales, SMS enables the synthesis of nanostructured materials and catalysts. These include metal and metal-halide nanoparticles, nanocatalysts for biodiesel production, photocatalytic composites, and magnetic biochar systems with high pollutant degradation and adsorption efficiency.
5. Biological recycling pathways such as composting and vermiculture remain important baseline management strategies for SMS. These approaches stabilize organic matter and return nutrients to soil systems, supporting circular nutrient cycling, although they generally generate lower economic value compared with materials-oriented valorization routes.
6. The diversity of SMS valorization pathways highlights its adaptability across environmental, materials, and energy applications. Performance in these systems depends strongly on feedstock composition, fungal species, pretreatment history, and thermochemical processing conditions.

Despite promising laboratory-scale results, most studies remain at early technology readiness levels. Key challenges include feedstock variability, drying energy requirements, standardization of processing conditions, and scalability of activation or functionalization strategies. Techno-economic analysis (TEA) and life-cycle assessment (LCA) remain critically underdeveloped. Most studies report laboratory performance metrics without providing the energy balances, logistics assumptions, and system boundaries required for realistic economic and environmental evaluation. Future research should prioritize standardized reporting and system-level evaluation. Essential parameters include SMS composition, moisture content, pretreatment conditions, wet- and dry-basis yields, energy consumption, transportation logistics, and the fate of residues or spent sorbents. Integrating TEA/LCA-informed experimental design will be essential for translating laboratory innovations into scalable technologies. Harmonized reporting of energy and material balances will enable cross-study comparison and realistic assessment of commercialization potential. With coordinated advances in process optimization, system integration, and cross-sector collaboration, SMS can transition from a waste management liability into a reliable feedstock for high-value materials and circular bioeconomy applications.
